# First-trimester artemisinin derivatives and quinine treatments and the risk of adverse pregnancy outcomes in Africa and Asia: A meta-analysis of observational studies

**DOI:** 10.1371/journal.pmed.1002290

**Published:** 2017-05-02

**Authors:** Stephanie Dellicour, Esperança Sevene, Rose McGready, Halidou Tinto, Dominic Mosha, Christine Manyando, Stephen Rulisa, Meghna Desai, Peter Ouma, Martina Oneko, Anifa Vala, Maria Rupérez, Eusébio Macete, Clara Menéndez, Seydou Nakanabo-Diallo, Adama Kazienga, Innocent Valéa, Gregory Calip, Orvalho Augusto, Blaise Genton, Eric M. Njunju, Kerryn A. Moore, Umberto d’Alessandro, Francois Nosten, Feiko ter Kuile, Andy Stergachis

**Affiliations:** 1 Malaria Epidemiology Unit, Department of Clinical Sciences, Liverpool School of Tropical Medicine, Liverpool, United Kingdom; 2 Faculty of Medicine, Eduardo Mondlane University, Maputo, Mozambique; 3 Centro de Investigação em Saúde da Manhiça, Manhiça, Mozambique; 4 Shoklo Malaria Research Unit, Mahidol Oxford Tropical Medicine Research Unit, Faculty of Tropical Medicine, Mahidol University, Mae Sot, Thailand; 5 Centre for Tropical Medicine and Global Health, Nuffield Department of Clinical Medicine, University of Oxford, Oxford, United Kingdom; 6 Institut de Recherche en Sciences de la Santé/Centre Muraz, Bobo-Dioulasso, Burkina Faso; 7 Ifakara Health Institute, Rufiji, Tanzania; 8 Tropical Diseases Research Centre, Ndola, Zambia; 9 University Teaching Hospital of Kigali, University of Rwanda, Kigali, Rwanda; 10 Malaria Branch, Centers for Disease Control and Prevention, Atlanta, Georgia, United States of America; 11 Centre for Global Health Research, Kenya Medical Research Institute, Kisumu, Kenya; 12 Instituto de Salud Global de Barcelona, Barcelona, Spain; 13 Department of Pharmacy Systems, Outcomes and Policy, University of Illinois at Chicago, Chicago, Illinois, United States of America; 14 Swiss Tropical and Public Health Institute, Basel, Switzerland; 15 Infectious Diseases Service, Lausanne University Hospital, Lausanne, Switzerland; 16 School of Medicine, Copperbelt University, Ndola, Zambia; 17 Centre for Epidemiology and Biostatistics, Melbourne School of Population and Global Health, University of Melbourne, Melbourne, Victoria, Australia; 18 Macfarlane Burnet Institute for Medical Research and Public Health, Melbourne, Victoria, Australia; 19 Medical Research Council, Fajara, The Gambia; 20 Institute of Tropical Medicine, Antwerp, Belgium; 21 London School of Hygiene & Tropical Medicine, London, United Kingdom; 22 Department of Pharmacy, School of Pharmacy, University of Washington, Seattle, Washington, United States of America; 23 Department of Global Health, School of Public Health, University of Washington, Seattle, Washington, United States of America; St. George's, University of London, UNITED KINGDOM

## Abstract

**Background:**

Animal embryotoxicity data, and the scarcity of safety data in human pregnancies, have prevented artemisinin derivatives from being recommended for malaria treatment in the first trimester except in lifesaving circumstances. We conducted a meta-analysis of prospective observational studies comparing the risk of miscarriage, stillbirth, and major congenital anomaly (primary outcomes) among first-trimester pregnancies treated with artemisinin derivatives versus quinine or no antimalarial treatment.

**Methods and findings:**

Electronic databases including Medline, Embase, and Malaria in Pregnancy Library were searched, and investigators contacted. Five studies involving 30,618 pregnancies were included; four from sub-Saharan Africa (*n* = 6,666 pregnancies, six sites) and one from Thailand (*n* = 23,952). Antimalarial exposures were ascertained by self-report or active detection and confirmed by prescriptions, clinic cards, and outpatient registers. Cox proportional hazards models, accounting for time under observation and gestational age at enrollment, were used to calculate hazard ratios. Individual participant data (IPD) meta-analysis was used to combine the African studies, and the results were then combined with those from Thailand using aggregated data meta-analysis with a random effects model.

There was no difference in the risk of miscarriage associated with the use of artemisinins anytime during the first trimester (*n* = 37/671) compared with quinine (*n* = 96/945; adjusted hazard ratio [aHR] = 0.73 [95% CI 0.44, 1.21], *I*^2^ = 0%, *p* = 0.228), in the risk of stillbirth (artemisinins, *n* = 10/654; quinine, *n* = 11/615; aHR = 0.29 [95% CI 0.08–1.02], *p* = 0.053), or in the risk of miscarriage and stillbirth combined (pregnancy loss) (aHR = 0.58 [95% CI 0.36–1.02], *p* = 0.099). The corresponding risks of miscarriage, stillbirth, and pregnancy loss in a sensitivity analysis restricted to artemisinin exposures during the embryo sensitive period (6–12 wk gestation) were as follows: aHR = 1.04 (95% CI 0.54–2.01), *I*^2^ = 0%, *p* = 0.910; aHR = 0.73 (95% CI 0.26–2.06), *p* = 0.551; and aHR = 0.98 (95% CI 0.52–2.04), *p* = 0.603. The prevalence of major congenital anomalies was similar for first-trimester artemisinin (1.5% [95% CI 0.6%–3.5%]) and quinine exposures (1.2% [95% CI 0.6%–2.4%]). Key limitations of the study include the inability to control for confounding by indication in the African studies, the paucity of data on potential confounders, the limited statistical power to detect differences in congenital anomalies, and the lack of assessment of cardiovascular defects in newborns.

**Conclusions:**

Compared to quinine, artemisinin treatment in the first trimester was not associated with an increased risk of miscarriage or stillbirth. While the data are limited, they indicate no difference in the prevalence of major congenital anomalies between treatment groups. The benefits of 3-d artemisinin combination therapy regimens to treat malaria in early pregnancy are likely to outweigh the adverse outcomes of partially treated malaria, which can occur with oral quinine because of the known poor adherence to 7-d regimens.

**Review registration:**

PROSPERO CRD42015032371

## Abstract 2

In a meta-analysis using individual patient data from prospective observational studies, Andy Stergachis and colleagues investigate the risk of of miscarriage, stillbirth, and major congenital anomaly among pregnancies that were treated during the first trimester with artemisinin derivatives versus quinine or no antimalarial treatment.

## Author summary

**Why was this study done?:**

- Malaria infection is more frequent and severe in pregnant women compared to non-pregnant women, and the adverse consequences of malaria in pregnancy require prompt, safe, and effective treatment.
- Artemisinin combination therapies (ACTs), the most efficacious antimalarials available, are the recommended first-line treatment for *Plasmodium falciparum* malaria.
- Animal embryotoxicity data and the paucity of safety data in human pregnancies have prevented artemisinin derivatives from being recommended for malaria treatment in the first trimester except in life-saving circumstances, yet in malaria endemic countries, many early pregnancies are advertently or inadvertently exposed to ACTs.
- In this meta-analysis of prospective observational studies, we summarize all available safety data on the effect of artemisinin exposure in the first trimester and compare the risk of miscarriage, stillbirth, and major congenital anomaly for pregnancies treated with artemisinin, quinine, or no antimalarials in the first trimester.

**What did the researchers do and find?:**

- Our team determined the risk of miscarriages, stillbirths, and major congenital anomalies associated with first-trimester artemisinin treatment versus quinine in four independent prospective observational studies across six sites in sub-Saharan Africa using individual participant data meta-analysis. The results were then combined with summary effect estimates from the Shoklo Malaria Research Unit on the Thailand–Myanmar border using aggregated data meta-analysis.
- We found no difference in the risk of miscarriage, stillbirth, or major congenital anomalies associated with the use of artemisinins anytime during the first trimester compared with the use of quinine during the same gestational period.

**What do these findings mean?:**

- The ACT class of antimalarials should be considered for treatment of malaria in the first trimester of pregnancy.
- The limited data on the risk of congenital anomalies require further observational studies.

## Introduction

Artemisinin combination therapies (ACTs), the most efficacious antimalarials available, are the recommended first-line treatment for *Plasmodium falciparum* malaria except in the first trimester of pregnancy [1]. Preclinical studies have demonstrated that artemisinin derivatives are embryotoxic and can induce fetal death and congenital anomalies at doses close to the therapeutic range in multiple animal species [2–10]. In rodents, artemisinins cause embryolethality as well as cardiovascular (ventricular septal and vessel defects) and skeletal defects (shortened or bent long bones and scapulae, misshapen ribs, cleft sternebrae, and incompletely ossified pelvic bones) [6]. In monkeys, embryolethality was observed following prolonged treatment (12 to 20 d), but there were no malformations [2,4]. The artemisinin embryotoxic effect occurs through depletion of embryonic erythroblasts [11]. It is unknown how findings from animal studies would translate in humans because the mechanism of teratogenicity and the drug sensitive period may differ significantly in humans [3,12]. The last review by the World Health Organization (WHO) dates from 2006, when evidence on 170 human first-trimester artemisinin treatments was reassuring but insufficient to inform policy change [13]. Consequently, quinine remains the recommended treatment for uncomplicated *P*. *falciparum* malaria in the first trimester. Presently, artemisinins are recommended in the first trimester only if quinine cannot be used or in cases of severe malaria where the benefit outweighs the potential risk.

Weighing the risks and benefits of artemisinin treatment in the first trimester is important for public health policy as well as for individual treatment decisions. Malaria is more frequent and severe in pregnant women compared to non-pregnant women [14–16]. Because of the adverse consequences of malaria in pregnancy, prompt, safe, and effective treatment is required [17,18]. Concerns over the efficacy, tolerance, adherence, and availability of quinine calls for a reconsideration of its risk–benefit balance relative to ACTs [19,20]. Malaria infection in the first trimester leads to placental infection associated with maternal anemia, low birth weight, and intrauterine growth retardation [17,21,22]. First-trimester malaria documented on the Thailand–Myanmar border was strongly associated with miscarriage, especially following recurrence of infection [23,24]. Malaria-associated risks must be balanced with any safety risks due to treatment. The assessment of treatment risk during pregnancy is complex and requires detection and confirmation of (1) malaria, (2) antimalarial treatment in the first trimester, and (3) adverse outcomes, and this complexity has implications for the management of confounding factors in statistical analyses. Case–control studies can be useful and efficient for assessing drug safety signals; however, this approach is not recommended for pregnancy outcomes due to the high risk of recall bias [25].

To compare the potential harm versus beneficial impact on pregnancy outcomes of treatment with artemisinin derivatives versus quinine, we determined the risk of miscarriage, stillbirths, and major congenital anomalies associated with first-trimester artemisinin treatment in four independent prospective observational studies across six sites in sub-Saharan Africa using individual participant data (IPD) meta-analysis [26] and combined the results with summary effect estimates from the Shoklo Malaria Research Unit (SMRU) at the Thailand–Myanmar border, using aggregated data meta-analysis.

## Methods

### Ethics

All studies had institutional ethical review approvals and obtained informed consent from all participants. The institutional ethics review committees for the individual studies are listed in S2 Text.

### Search strategy

The protocol for this meta-analysis was registered on PROSPERO (CRD42015032371). We report our findings in accordance with the Preferred Reporting Items for Systematic Reviews and Meta-Analyses (PRISMA) checklist of items specific to IPD meta-analyses (S1 Checklist) [27].

We conducted an electronic search of Medline, Embase, and the Malaria in Pregnancy Library as of November 16, 2015, using the PICOTS (patient, intervention, comparator, outcome, timing, and setting) framework (S1 Text) [28]. We also searched trial registries, “gray literature” databases, and conference abstracts; manually reviewed reference lists of selected publications; and contacted experts in the field to get information on unpublished studies.

### Inclusion/exclusion criteria

Studies were eligible if (1) the study reported artemisinin derivative use in the first trimester of pregnancy, (2) antimalarial treatment was confirmed through multiple data sources, (3) women were enrolled before pregnancy outcome was known (i.e., prospective follow-up of pregnancy), and (4) the study included internal comparison groups with either quinine treatment or no antimalarial treatment in the first trimester. Studies were excluded if they were case series, retrospective or case–control studies, or cross-sectional surveys; did not involve artemisinins treatment; reported only second and third trimester artemisinin exposures; or did not report pregnancy outcomes (S3 Text).

### Data extraction and compilation

The first author or principal investigator of each study was invited to participate in the pooled analysis and was asked to provide individual-level data (S1 Text). The data requested included relevant baseline characteristics, gestational age, antimalarial exposure information, pregnancy outcomes, and congenital anomalies.

### Risk of bias assessment

The Newcastle–Ottawa scale for assessing bias in cohort studies was used [29] to assess the IPD studies with respect to the selection of the artemisinin, quinine, and untreated groups; the comparability of the groups; and the ascertainment of the outcomes of interest, including loss to follow-up (see S2 Text for ratings of each study for the IPD analysis).

### Antimalarial exposure group definitions

Exposures were considered confirmed if information could be verified across at least two data sources (self-reported by the woman and confirmed from clinic cards, outpatient registers, or prescription sheets or directly observed/documented by concurrent independent surveillance in Kenya [30]). Otherwise, treatments were classified as unconfirmed and excluded from the analysis. The treatments of interest were artemisinin derivatives or quinine in the first trimester of pregnancy (≤13 wk from the date of the last menstrual period). For the IPD studies, pregnancies were considered unexposed if there was no evidence (including unconfirmed exposures) of antimalarial treatment up to 18 wk gestation; in the SMRU analysis, unexposed pregnancies were pregnancies without microscopically confirmed malaria and without antimalarial treatment in the first trimester. Further details on the exposure definitions used for the IPD and SMRU analyses are provided in S3 Text.

### Outcome definitions

The primary outcomes of interest were (1) miscarriage, defined as a confirmed pregnancy (identified by pregnancy test or ultrasound and/or examination at antenatal care visit) that ended at or before 28 wk gestation; (2) stillbirth, defined as fetal death after 28 wk gestation in utero or during labor, and (3) major congenital anomaly, defined as any structural abnormality with surgical, medical, or cosmetic importance detected by surface examination at birth. In addition, cases with two or more minor anomalies were considered as major congenital anomaly for the African sites, as per the Antiretroviral Pregnancy Registry guidelines. An expert birth defect panel established by the WHO Pregnancy Registry pilot project [31], the WHO Birth Defect Panel, independently and blinded to exposure status, reviewed and classified all the congenital anomalies from three sites under a multicenter study protocol, the Assessment of the Safety of Antimalarial Drug Use during Early Pregnancy (ASAP) study. For the other studies, criteria to differentiate between major and minor anomalies and to determine anomalies for exclusion, such as genetic and chromosomal disorders, were applied based on the WHO Birth Defect Panel recommendations and published criteria [32]. Data from Rwanda were not included in the analysis of congenital anomalies as only major anomalies were reported, and two out of the six anomalies were detected in participants enrolled after delivery.

### Data analysis

The data analysis was prespecified in an analytical plan (S1 Text). Statistical analyses were performed in Stata version 13.1, R version 3.2.1, and SAS version 9.4. Further details on statistical methods are provided in S4 Text.

The primary analysis compared pregnancies with a confirmed treatment with artemisinin to pregnancies with a confirmed treatment with quinine. A secondary analysis compared miscarriage, stillbirth, and major congenital anomaly in women treated with artemisinin or quinine to those in women who did not have malaria in the first trimester. In Africa, it was assumed that a woman did not have malaria if she did not receive antimalarials; in Asia, not having malaria was microscopically confirmed by repeated screens.

### Individual participant data check and missing data

Data were re-coded as necessary to create a pooled dataset for analysis purposes. The integrity of the data was checked by comparing the data in published reports with the data provided, as well as through review of internal out-of-range inconsistencies. In case of discrepancies, the investigators were contacted for clarification. Women for whom pregnancy outcome was unknown were considered lost to follow-up. These women were included in the analysis and contributed person-time until the last visit date when they were censored. Missing values were included as “unknown” categorically.

### Individual participant data statistical approach

**Fig 1:**
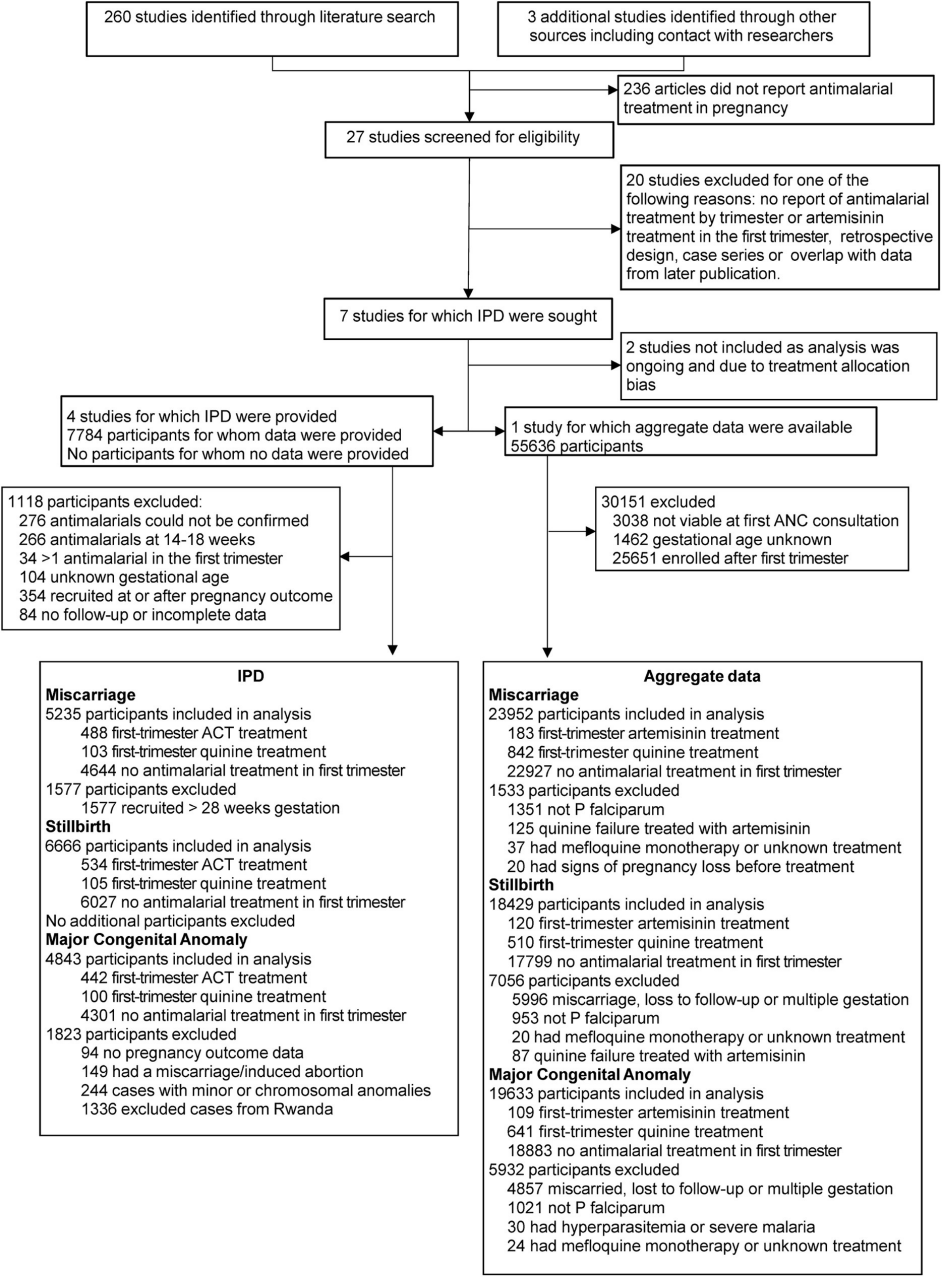
Flow diagram of studies and participants included in meta-analysis for miscarriage, stillbirth, and congenital anomaly. ACT, artemisinin combination therapy; ANC, antenatal care; IPD, individual participant data.

IPD estimates were for women who received first-line quinine or first-line artemisinin treatment for uncomplicated falciparum malaria in the first trimester, and excluded women with unknown gestational age, women who received more than one antimalarial treatment in the first trimester, those recruited at or after pregnancy outcome, and those with an unconfirmed antimalarial exposure (Fig 1).

For the miscarriage and stillbirth endpoints, hazard ratios (HRs) and 95% confidence intervals (CIs) were estimated using Cox regression models to account for time under observation and different gestational age at enrollment. Exposure was treated as time-dependent so that participants were considered exposed only from the time they received artemisinin or quinine treatment rather than from the time of enrollment in the study. The estimated log HRs were combined across studies using one-stage random effects meta-analysis adjusting for clustering by study site, along with assessment of heterogeneity. Heterogeneity across studies was assessed using *I*^2^ for both one-stage and two-stage IPD analysis. The proportional hazards assumptions of the Cox model were evaluated by testing for interactions between our time-varying exposures of interest and the logarithm of follow-up time, and further examined using log (−log[survival]) versus log of survival time plots. To account for the correlation between pregnancies for women with more than one pregnancy within a study, a robust variance estimator was used. All crude effect estimates in the IPD analysis were site-adjusted. In addition we conducted multivariate analyses adjusting for potential confounders identified a priori and available from all sites (gravidity and age). Adjusted models do not include HIV status as it is likely missing not at random with respect to antimalarial treatment and miscarriage (missing for 8% of participants and for 40% of those with a miscarriage). Descriptive statistics were used to classify congenital anomalies using the Antiretroviral Pregnancy Registry Organ System Classification [33]. Pooled prevalence of major congenital anomaly and its 95% confidence interval were estimated through inverse weighting of variance and random effects, with 0.05 continuity correction for zero count frequencies.

### Aggregated data meta-analysis

For miscarriage and stillbirth, aggregated data were pooled and combined into summary HRs using a Mantel–Haenszel random effects model combining the IPD analysis from the African sites with effect estimates from Asia. Effect estimates from the SMRU were based on a similar Cox regression model accounting for left truncation and treating antimalarial treatment as time-dependent as described in a recently published report [24]. SMRU estimates were for women who presented to antenatal clinics during their first trimester with a viable fetus and included uncomplicated, hyperparasitemic, and severe malaria and pregnancies with multiple malaria episodes. Multivariate analyses adjusted for infection severity (asymptomatic, symptomatic, hyperparasitemic/severe), year of first consultation, and non-malaria febrile morbidity in first trimester. HIV was not accounted for, but the prevalence on the Thailand–Myanmar border is very low [34]. Count data on major congenital anomalies by organ system were used to derive pooled prevalence estimates for exposure groups. A summary of inclusion and exclusion criteria for the SMRU and for the African site IPD is provided in S3 Text.

### Sensitivity analysis

Animal reproductive toxicity studies suggest that the embryo sensitive period in humans for artemisinins is between 4 and 10 wk post-conception (6–12 wk after last menstrual period) [11,12]. Therefore, we conducted sensitivity analyses to explore the effect of restricting the analysis to treatments that occurred in the embryo sensitive period for artemisinin. The effect size should be highest for treatments restricted to that embryo sensitive period if the embryotoxicity mechanism hypothesized from animal models applies to humans. Additional sensitivity analyses were performed to assess (1) the robustness of the results when using a two-stage IPD meta-analysis approach, (2) removing one site at a time from the meta-analysis and testing the effect of each study on the pooled estimates, and (3) the effect of imputation and stratified analysis by HIV status given the high proportion of miscarriage cases with unknown HIV status in the IPD from the African sites.

### Post hoc multivariate network meta-analysis

Following a suggestion by one of the reviewers, we also conducted multivariate network meta-analysis to evaluate the combined effect of artemisinins on miscarriage and stillbirth (pregnancy loss) while accounting for the correlation structure between these two outcomes [35,36] (see S4 Text).

## Results

Twenty-seven studies were identified, of which seven were eligible and five were included in the analysis: four from sub-Saharan Africa and one from the SMRU in Thailand [23] (Figs 1 and S1). The four African studies included a study conducted in three sites under a single multicenter study protocol (ASAP study) [35] and three additional stand-alone studies [36–38].

The excluded studies included one multi-country study coordinated by WHO [31] for which the analysis was still ongoing and one prospective cohort study from Indonesia [39] that met the inclusion criteria but was excluded due to systematic bias by indication: dihydroartemisinin-piperaquine was prescribed selectively for the sicker patients, as reported in the published paper.

### Characteristics of the individual participant data studies

All contacted investigators agreed to participate and sent the complete, anonymized individual-level data of their respective studies following completion of a data sharing agreement. Data from four prospective cohort studies contributed to the IPD meta-analysis, with the number of pregnancies included in the analysis depending on the outcome (Table 1). The studies were conducted in six countries in sub-Saharan Africa (Zambia, Tanzania, Rwanda, Kenya, Mozambique, and Burkina Faso) between 2004 and 2013. Description of participant characteristics across exposure categories for participants included in the IPD analysis is presented in Table 2. For discrepancies between data included in this IPD analysis and published reports, see S5 Text.

**Table 1:** Description of prospective cohort studies and data included in the African individual participant data meta-analysis and Asian aggregated data meta-analysis.

Study	Study site	Study period	Mean gestational age at enrollment (SD)	Mean gestational weeks of follow-up (SD)	Number of confirmed first trimester artemisinin treatments	Number of confirmed first-trimester quinine treatments	Number with no antimalarials in first trimester^a^	Number of miscarriages	Number of stillbirths	Number of live births
**Africa**										
Manyando et al. [36]	Zambia	2004–2008	25.1 (8.1)	13.7 (8.1)	179	4	632	9	17	754
Rulisa et al. [38]	Rwanda	2007–2009	26.9 (8.0)	11.4 (7.6)	77	0	1,515	12	47	1,533
Mosha et al. [37]	Tanzania	2012–2013	14.6 (3.5)	20.1 (11.1)	156	69	1,533	41	61	1,656
Dellicour et al. [30]; Tinto et al [35]	Kenya^b^	2011–2013	15.5 (8.9)	20.4 (10.7)	64	3	993	62	23	880
Tinto et al. [35]	Mozambique^b^	2011–2013	21.0 (5.7)	17.8 (10.3)	24	5	721	13	19	691
Tinto et al. [35]	Burkina Faso^b^	2011–2013	23.2 (6.8)	14.6 (6.3)	34	24	632	6	13	671
Total all IPD		2004–2013	20.8 (8.7)	15.1 (9.3)	534^c^	105	6,027	143	180	6,185
**Asia**										
Moore [24]	Thailand–Myanmar border	1994–2013	9.0 (2.6)		183^d^	842	22,927	2,257	185	18,537
**Total all studies**	**Africa and Asia**	**1994–2013**	**11.5 (3.5)**		**717**	**947**	**28,954**	**2,400**	**365**	**24,722**

All women were recruited prospectively before pregnancy outcome was known, but a combination of prospective and retrospective approaches were used to assess antimalarial exposure information. The numbers represent the total number of enrolled pregnancies; however, varying inclusion/exclusion criteria were applied for the analyses of the various outcomes, and the numbers vary accordingly.

^a^Unexposed to any antimalarial up to gestational week 18 for the African sites and up to gestational week 14 for the Thailand–Myanmar border.

^b^These three sites were part of a multicenter study, the ASAP study, coordinated by the Malaria in Pregnancy Consortium, using a standard protocol and with a planned IPD analysis.

^c^Artemisinin treatment: 501 artemether-lumefantrine and 33 artesunate-amodiaquine (Burkina Faso).

^d^Artemisinin treatment: 71 mefloquine-artesunate, 50 artesunate-clindamycin, 49 artesunate monotherapy, 10 artemether-lumefantrine, and 3 dihydroartemisinin-piperaquine.

IPD, individual participant data; SD, standard deviation.

**Table 2:** Descriptive characteristics of pregnancies across exposure categories for the African sites contributing to the individual patient data meta-analysis.

Characteristic	All pregnancies, *n* = 6,666	No antimalarial use first trimester, *n* = 6,027	Confirmed ACT use first trimester, *n* = 534	Confirmed quinine use first trimester, *n* = 105
**Age (years)**				
Mean (SD)	26.1 (6.4)	26.1 (6.4)	25.9 (6.2)	25.6 (5.9)
<20 y	1,071 (16.1)	975 (16.2)	76 (14.2)	20 (19.1)
20–24 y	1,961 (29.4)	1,754 (29.1)	180 (33.7)	27 (25.7)
25–29 y	1,697 (25.5)	1,541 (25.6)	122 (22.9)	34 (32.4)
30+ y	1,937 (29.0)	1,757 (29.2)	156 (29.2)	24 (22.9)
**Gravidity**				
Primigravida	1,695 (25.4)	1,505 (25.0)	154 (28.8)	36 (34.3)
1–3 pregnancies	3,269 (49.0)	2,964 (49.2)	255 (47.8)	50 (47.6)
4+ pregnancies	1,570 (23.6)	1,429 (23.7)	124 (23.2)	17 (16.2)
Missing	132	129	1	2
**Marital status**				
Single	828 (12.4)	768 (12.7)	51 (9.6)	9 (8.6)
Married or living together	3,340 (50.1)	3,031 (50.3)	218 (40.8)	91 (86.7)
Missing	2,498	2,228	265	5
**Education**				
Primary not completed	1,583 (23.8)	1,464 (24.3)	92 (17.2)	27 (25.7)
Primary completed	2,475 (37.1)	2,183 (36.2)	236 (44.2)	56 (53.3)
Secondary completed	944 (14.2)	799 (13.3)	124 (23.2)	21 (20.0)
Missing	1,664	1,581	82	1
**HIV status**				
Negative	5,694 (85.4)	5,125 (85.0)	471 (88.2)	98 (93.3)
Positive	567 (8.5)	528 (8.8)	37 (6.9)	2 (1.9)
Missing	405	374	26	5
**Gestational age in weeks at enrollment**				
Mean (SD)	20.8 (8.7)	21.4 (8.6)	15.2 (7.7)	14.6 (5.1)
Median (IQR)	19 (14–27)	20 (16–28)	13 (10–19)	14 (12–17)
**Duration of follow-up in weeks**	26.1			
Mean (SD)	15.1 (9.3)	14.8 (9.3)	18.2 (8.9)	22.1 (6.6)
Median (IQR)	14 (8–20)	13 (8–20)	18 (11–25)	23 (19–26)

Data are *n* (percent) unless otherwise indicated.

ACT, artemisinin combination therapy; IQR, interquartile range; SD, standard deviation.

### Inclusion of data from Asia in the aggregated data meta-analysis

Aggregated data from the SMRU was included for the assessment of miscarriages, stillbirths, and major congenital anomalies (detailed description of the site and methods is published in [24]). There were no stillbirths in the women treated with artemisinin in the first trimester, and therefore there are no SMRU estimates for that group.

### Miscarriage

**Fig 2:**
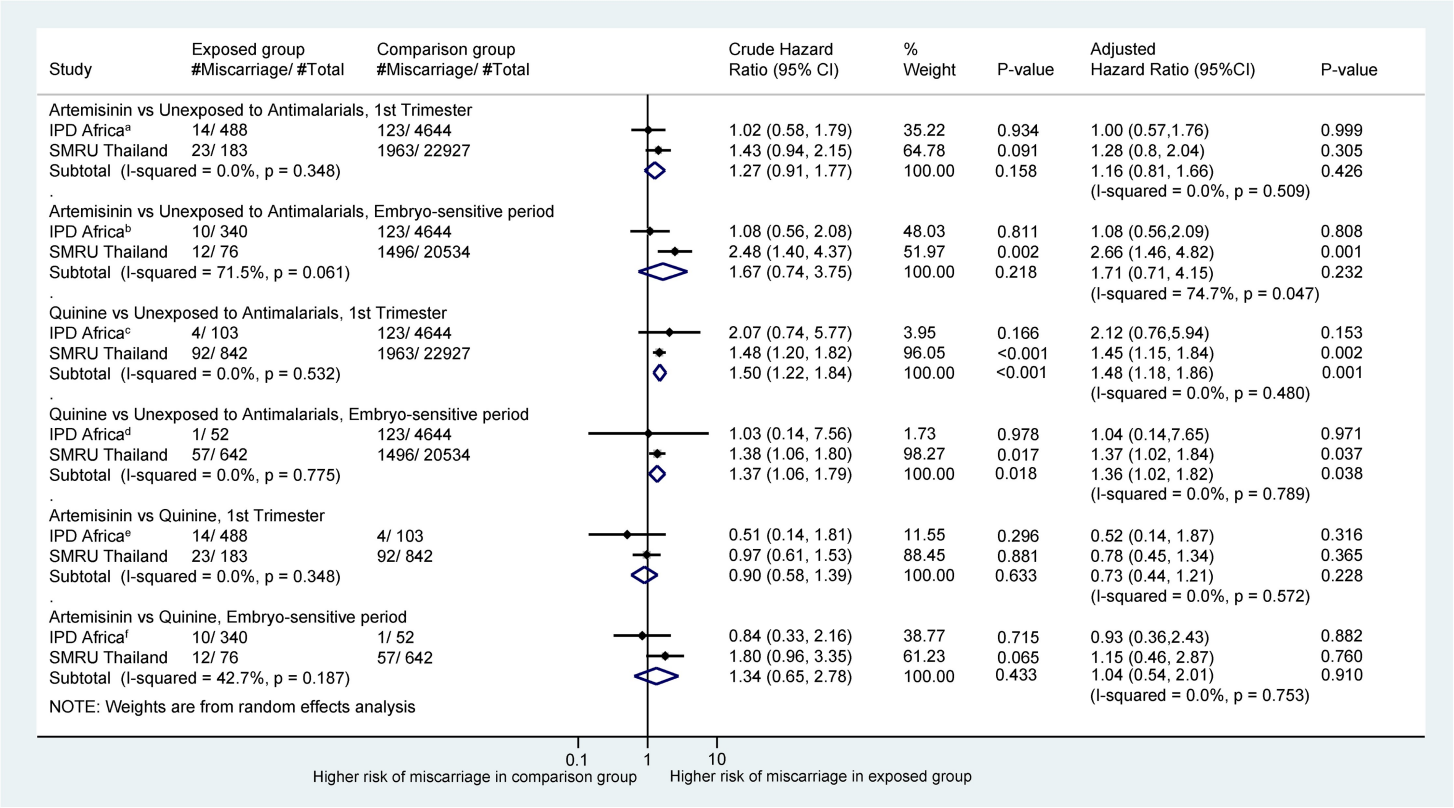
Forest plot for aggregated data meta-analysis of crude and adjusted hazard ratios for the association between different antimalarial exposure categories and miscarriage. HRs account for pregnancy week under observation through left truncation and treat exposure as a time-dependent variable. *I*^2^ values are for IPD from Africa only: ^a^26.4%; ^b^26.0%; ^c^23.9%; ^d^30.5%; ^e^26.1%; ^f^28.9%. Crude HRs are adjusted for clustering by site in the IPD arm. aHRs for IPD from Africa account for site, woman’s age, and gravidity. aHRs for the SMRU account for smoking, year of first consultation, gravidity, and non-malaria febrile morbidity in first trimester for the comparison with the unexposed group, while the comparison between the artemisinin and quinine groups accounts for infection severity (asymptomatic, symptomatic, hyperparasitemic/severe), year of first consultation, and non-malaria febrile morbidity in the first trimester. aHR, adjusted hazard ratio; HR, hazard ratio; IPD, individual patient data; SMRU, Shoklo Malaria Research Unit.

Pregnancies treated with an artemisinin derivative in the first trimester were at a similar risk of miscarriage compared to those treated with quinine in the same period (exposure anytime in the first trimester: adjusted HR [aHR] = 0.73 [95% CI 0.44, 1.21], *I*^2^ = 0%; exposure during artemisinin embryo sensitive period: aHR = 1.04 [95% CI 0.54, 2.01], *I*^2^ = 0%; Fig 2). Pregnancies treated with quinine during the first trimester were associated with significantly increased risk of miscarriage compared with pregnancies not treated with an antimalarial (aHR = 1.48 [95% CI 1.18, 1.86]). Pregnancies treated with artemisinins during the first trimester were not associated with an increased risk of miscarriage compared with pregnancies not treated with an antimalarial (aHR = 1.16 [95% CI 0.81–1.66]; Fig 2).

### Stillbirth

**Fig 3:**
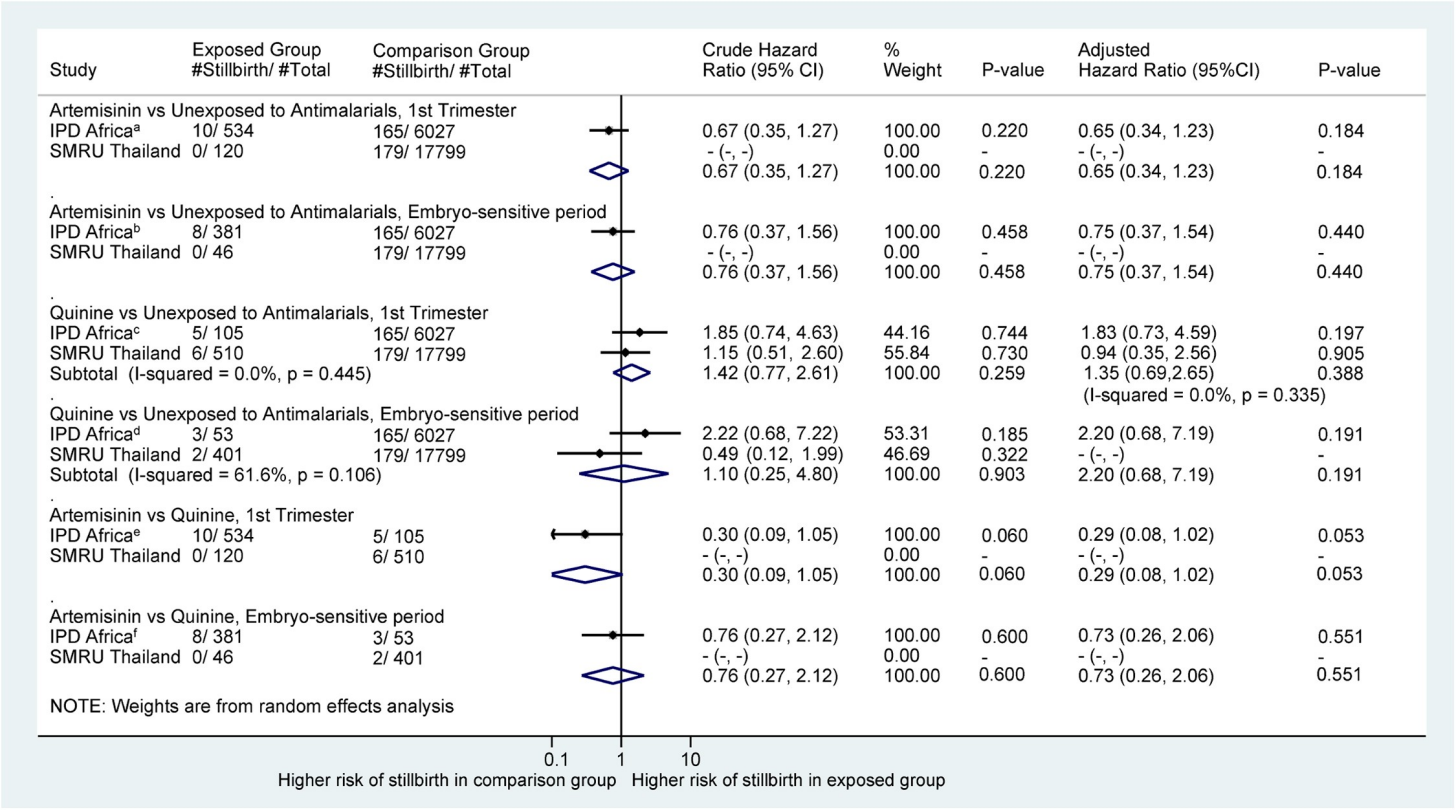
Forest plot for aggregated data meta-analysis of crude and adjusted hazard ratios for the association between different antimalarial exposure categories and stillbirth. HRs account for pregnancy week under observation through left truncation and treat exposure as a time-dependent variable. *I*^2^ values are for IPD from Africa only: ^a^26.0%; ^b^27.4%; ^c^26.1%; ^d^28.3%; ^e^20.5%; ^f^27.0%. Crude HRs are adjusted for clustering by site. aHRs for IPD from Africa account for site, woman’s age, and gravidity. aHRs for the SMRU account for smoking, year of first consultation, gravidity, and non-malaria febrile morbidity in the first trimester for the comparison with the unexposed group. aHR, adjusted hazard ratio; HR, hazard ratio; IPD, individual patient data; SMRU, Shoklo Malaria Research Unit.

There were no differences in the risk of stillbirth for pregnancies treated with an artemisinin compared to those treated with quinine anytime in the first trimester or during the embryo sensitive period (aHR = 0.29 [95% CI 0.08, 1. 02] and 0.73 [95% CI 0.26, 2.06], respectively; Fig 3). Neither treatment with an artemisinin nor quinine was associated with an increased risk of stillbirths compared to pregnancies without any antimalarial treatment in the first trimester: aHR = 0.65 (95% CI 0.34, 1.23) and 1.35 (95% CI 0.69, 2.65), respectively.

### Multivariate meta-analysis

Multivariate meta-analysis for the adverse outcomes of miscarriage and stillbirth (i.e., pregnancy loss) also demonstrated no significant differences in risk for pregnancies treated with artemisinins compared to those treated with quinine (anytime during the first trimester: aHR = 0.58 [95% CI 0.36, 1.02], *p* = 0.099; embryo sensitive period: aHR = 0.98 [95% CI 0.52, 2.04], *p* = 0.603).

### Congenital anomalies

The pooled prevalence of major congenital anomaly among children born to women exposed to artemisinin and quinine anytime in the first trimester was 1.5% (95% CI 0.6%, 3.5%), and 1.2% (95% CI 0.6%, 2.4%), respectively, and was 2.4% (95% CI 0.9%, 6.1%) and 1.5% (95% CI 0.8%, 3.0%), respectively, for those exposed during the embryo sensitive period. Pooled prevalence of major congenital anomaly was 0.7% (95% CI 0.4%, 1.2%) for pregnancies unexposed to any antimalarial during the first trimester (S6 Table). There was no difference in the distribution of congenital anomalies across organ system classes between treatment groups, but numbers were small. Details on the distribution of minor and major congenital anomalies are provided in Table 3.

**Table 3:** Summary of the distribution of minor and major congenital anomalies by Antiretroviral Pregnancy Registry Organ System Classification across exposure groups.

Anomalies	First trimester			Embryo sensitive period^a^		Total, *n* = 24,396
	No antimalarial, *n* = 23,104	Artemisinin, *n* = 551	Quinine, *n* = 741	Artemisinin, *n* = 387	Quinine, *n* = 569	
**Organ system class**						
Central nervous system	37 (0.16)	0 (0.00)	1 (0.13)	0 (0.00)	1 (0.18)	38 (0.16)
Face and neck	33 (0.14)	3 (0.54)	0 (0.00)	2 (0.52)	0 (0.00)	36 (0.15)
Cleft lip and/or palate	0 (0.00)	0 (0.00)	1 (0.13)	0 (0.00)	1 (0.18)	1 (0.00)
Heart–other defects	4 (0.02)	0 (0.00)	1 (0.13)	0 (0.00)	1 (0.18)	5 (0.02)
Circulatory system–other defects	13 (0.06)	0 (0.00)	0 (0.00)	0 (0.00)	0 (0.00)	13 (0.05)
Respiratory system	2 (0.01)	1 (0.18)	0 (0.00)	1 (0.26)	0 (0.00)	3 (0.01)
Upper gastrointestinal system	0 (0.00)	0 (0.00)	2 (0.27)	0 (0.00)	2 (0.35)	2 (0.01)
Gastrointestinal system unspecified	59 (0.26)	0 (0.00)	0 (0.00)	0 (0.00)	0 (0.00)	59 (0.24)
Female genitalia	3 (0.01)	1 (0.18)	0 (0.00)	1 (0.26)	0 (0.00)	4 (0.02)
Male genitalia	3 (0.01)	0 (0.00)	0 (0.00)	0 (0.00)	0 (0.00)	3 (0.01)
Genitalia unspecified	19 (0.08)	0 (0.00)	0 (0.00)	0 (0.00)	0 (0.00)	19 (0.08)
Renal and urinary system	7 (0.03)	0 (0.00)	0 (0.00)	0 (0.00)	0 (0.00)	7 (0.03)
Limb reduction/addition defects	9 (0.04)	1 (0.18)	0 (0.00)	1 (0.26)	0 (0.00)	10 (0.04)
Musculoskeletal–other defects	59 (0.26)	1 (0.18)	4 (0.54)	0 (0.00)	4 (0.70)	64 (0.26)
Skin and skin derivatives	7 (0.03)	2 (0.36)	0 (0.00)	2 (0.52)	0 (0.00)	9 (0.04)
Other organs and organ systems	1 (0.00)	0 (0.00)	0 (0.00)	0 (0.00)	0 (0.00)	1 (0.00)
Unspecified	26 (0.11)	0 (0.00)	1 (0.13)	0 (0.00)	1 (0.18)	27 (0.11)
**Total anomalies included in the analysis**	282 (1.22)	9 (1.63)	10 (1.35)	7 (1.81)	10 (1.76)	301 (1.23)
**Babies with at least two minor anomalies**	40 (0.17)	3 (0.54)	0 (0.00)	2 (0.52)	0 (0.00)	43 (0.18)
**Babies with major anomalies (not including two minor anomalies)**	176 (0.76)	2 (0.36)	8 (1.08)	2 (0.52)	8 (1.41)	186 (0.76)
**Babies with major anomalies (major or two minor anomalies)**	187 (0.81)	5 (0.91)	8 (1.08)	4 (1.03)	8 (1.41)	200 (0.82)

Exclusion and inclusion criteria were based on the WHO Birth Defect Panel. Babies were assessed by surface exams, and functional or internal defects requiring additional test/examination could not be detected unless obvious without such examination.

^a^The putative embryo sensitive weeks for artemisinin extrapolated from animal data. There is no suspected embryo sensitive period for quinine.

### Sensitivity analysis

Results from the sensitivity analysis are available in S1–S4 Figs and S1 Table. Results from the two-stage IPD meta-analysis for the African sites showed effect estimates for both miscarriage and stillbirth similar to those from the one-stage IPD meta-analysis, although with slightly higher HRs and wider confidence intervals as certain sites no longer contributed data due to non-events. No difference between treatment groups was observed in this two-stage IPD analysis, as for the one-stage IPD analysis (first-trimester ACT versus quinine: aHR = 0.64 [95% CI 0.14, 3.04], *p* = 0.577, for miscarriage and aHR = 0.12 [95% CI 0.01, 3.02], *p* = 0.196, for stillbirth; S2 Fig and S3 Fig, respectively). The sensitivity analysis examining the effect of removing one study site at a time on the overall aggregated effect estimates showed that the effect estimates were stable, with minimal variation due to exclusion of individual sites (aHRs for first-trimester ACT versus quinine ranged from 0.51 when Thailand data were omitted to 0.94 when Tanzania data were omitted, and none were statistically different; S4 Fig). The association between miscarriage and first-trimester quinine treatment compared with no antimalarial treatment was no longer significant when data from Thailand were omitted (aHR = 2.12 [95% CI 0.76, 5.94], *p* = 0.153). Effect estimates from models adjusting for HIV status in the African site IPD—by including individuals with missing HIV status in an “unknown” category or by using multiple imputation—were similar (within 15%) to the effect estimate adjusting for age and gravidity only for the primary analysis (comparison between artemisinin and quinine treatment) (first-trimester ACT versus quinine: aHR = 0.55 [95% CI 0.15, 2.02], *p* = 0.370, for the model including HIV as a categorical variable with unknown and aHR = 0.57 [95% CI 0.15, 2.15], *p* = 0.335, for the model with imputed missing HIV data; S1 Table).

## Discussion

To our knowledge, this meta-analysis provides the most comprehensive and up-to-date analysis of the potential effects of inadvertent or intentional treatment with artemisinin derivatives in the first trimester of pregnancy and includes results from 1,664 well-documented pregnancies followed prospectively after artemisinin (717) or quinine (947) treatment in the first trimester.

We found no evidence that the artemisinin-associated embryotoxicity observed in cross-species animal models (embryolethality and congenital anomalies [3]) was present in human pregnancies. The available data provide no evidence of an increased risk of miscarriage or stillbirth among pregnancies with a confirmed first-trimester artemisinin treatment compared to pregnancies with quinine or no antimalarial treatment. Restricting exposures to the hypothesized artemisinin embryo sensitive period also indicates no difference in pregnancy loss between artemisinin and quinine treatment, although the number of women and events contributing to this secondary analysis was smaller and the available data can exclude only an increase in risk of miscarriage greater than 2.0-fold (Fig 2) and an increase in risk of stillbirth greater than 2.1-fold (Fig 3). These results were supported by the post hoc multivariate network meta-analysis of pregnancy loss that combined the results of miscarriage and stillbirth into a single effect estimate. The results for major congenital anomalies should be interpreted with caution due to small numbers of events. Nevertheless, the data so far indicate no difference in the prevalence of major congenital anomalies between treatment groups.

Consistent with the known adverse effects of malaria in pregnancy [17,18,22,23], our results indicate that pregnancies treated with quinine in early pregnancy were associated with an increased risk of miscarriage compared to pregnancies not requiring treatment for malaria. There was no such significant association for women treated with an artemisinin. This secondary analysis should be interpreted with caution as it is likely confounded by malaria, which is itself a risk factor for miscarriage (i.e., confounding by indication). It is possible that the increased risk of miscarriage associated with quinine reflects inadequately treated malaria resulting from the known drawbacks of oral quinine, including a lengthy 7-d treatment course with three-times-daily dosing, low tolerability, and associated poor compliance [19,40–42]. However, due to limited information on the malaria episodes being treated (including laboratory confirmation of malaria, parasitemia, and severity) from the African sites and unmeasured factors affecting health-seeking behaviors and clinician treatment decision-making, it is not possible to know whether such an effect is due to quinine or the severity of the underlying infection.

Our results suggest that the ACT class of antimalarials should be considered for treatment of malaria in the first trimester. ACTs remain the most effective antimalarials to date, are well tolerated, and are widely available. Trials in Southeast Asia, South America, and Africa found that artemisinins performed better than quinine for the treatment of severe malaria [43], uncomplicated malaria in non-pregnant adults [40], and malaria in the second and third trimester of pregnancy [19]. Quinine has been used since the 17th century [40] and is thought to be safe in pregnancy although there have not been any formal studies in early pregnancy assessing its safety and efficacy [44]. Though WHO recommends the combination of quinine and clindamycin, this combination is rarely used in practice due to high cost [45] and the low availability of clindamycin in malaria endemic countries. Our results also suggest that oral quinine, even as monotherapy, was not commonly used for first-trimester malaria in the African sites, and the number of documented quinine treatments was relatively small compared to treatment with artemether-lumefantrine. More studies are needed to understand the reasons behind this prescription pattern.

The power to exclude an increased risk of congenital anomalies with artemisinin treatment was limited in the current study. A sample size calculation suggested that 1,180 exposed cases (with a ratio of 1 exposed:4 unexposed and a background anomaly rate of 0.9%) would be enough to detect a doubling of risk of any major congenital anomaly detectable by surface examination. This is double the number of first-trimester artemisinin exposures presented in this paper (*n* = 551 for this endpoint). To exclude a 2-fold increase in the risk of a specific defect with an estimated background rate of 1/1,000, 10,748 first-trimester artemisinin treatments and 42,992 untreated pregnancies would be needed [46]. Such numbers will only be achievable using multiple sentinel sites over a decade and by pooling data in a global registry. Furthermore, no study has been designed to assess cardiovascular defects, which were detected as a potential problem in animal reprotoxicity studies in newborns, or any other internal defects due to the unavailability of appropriate equipment and expertise in the areas where the burden of malaria in pregnancy is highest.

A strength of this meta-analysis is the ability to standardize analyses across studies, including definitions of the embryo sensitive period, definitions of what constituted a confirmed treatment, and use of left truncation in the survival analysis for miscarriage [47] to avoid bias when gestational age at exposure and enrollment varied between comparison groups [48]. Several important limitations should be noted. We cannot account for potential confounding by indication nor by disease severity as these data were not available in the African studies. Information on malaria confirmation is important as malaria-infected cells could be at less risk of artemisinin toxicity [11]. It is therefore reassuring that the results from the adjusted models from Thailand, where this information was captured and women were recruited at earlier gestation overall, were similar to those of the African studies. While the artemisinin treatments from the African IPD analysis were predominantly artemether-lumefantrine (95%, 501/534) and the remaining 33 were artesunate-amodiaquine, all from Burkina Faso, artemisinin treatments from the SMRU included a wider range of regimens (mefloquine-artesunate, artemether-lumefantrine, artesunate-clindamycin, artesunate monotherapy, and dihydroartemisinin-piperaquine). This limits our ability to assess the effect of individual regimens, with the exception of artemether-lumefantrine. Evaluation of additional potential confounders was limited due to some missing data (e.g., previous pregnancy loss and HIV status), and potential for drug–drug interactions, such as between antimalarials and antiretrovirals, could not be assessed due to limited statistical power and incomplete information on antiretroviral treatment. Furthermore, dose–response effects could not be assessed due to the small numbers that had more than one artemisinin-based treatment in the first trimester (*n* = 5 for the SMRU and *n* = 9 across the African sites). Misclassification between miscarriage and stillbirth could have occurred due to errors in gestational age assessment and lack of data on the precise date of fetal death relative to date of expulsion/delivery. Furthermore, induced abortions could have been misclassified as miscarriage, but this is unlikely to differ by exposure group. Despite all sites having procedures in place to systematically detect external congenital anomalies at birth, the type of training, staff cadre, and timing of assessment may have affected the sensitivity of detection rate. Despite these limitations, the results reflect “real-life” effectiveness of artemisinin versus quinine, and the homogeneity of the findings across the studies provides further reassurance.

Despite the high burden of malaria in pregnancy, with an estimated 125 million at risk of infection annually in malaria endemic regions globally [49], fewer than 1,700 episodes of first-trimester quinine or artemisinin treatment have been documented prospectively with a known pregnancy outcome. In total, this has taken 32 years of cumulative and collective efforts since the introduction of artemisinins, including 13 years for 639 treatments in Africa and 19 years for 1,025 treatments in Asia. Up to now, conducting randomized controlled trials comparing ACTs to quinine for confirmed malaria in the first trimester of pregnancy was considered unethical; therefore, observational studies were the only available approach to monitor the safety of artemisinin treatment in early pregnancy. We have now reached a point of equipoise, so such a trial could be considered; however, randomized trials would not provide the sample size required to be informative on the risk of congenital anomalies. No single method can capture all desired data needed to make appropriate risk–benefit assessments of a drug used in pregnancy. A combination of different methods and data sources is the only feasible approach to gather the most complete picture of the potential developmental toxicity of a drug. The numbers required for the assessment of congenital anomalies could only be achieved through the combination of data across multiple sites. Identification of sentinel sites able to capture reliable data on drug exposure and pregnancy outcomes through a standard protocol is essential for safety signal detection and characterization [31,46]. Such an approach could be used for the evaluation of a wide variety of medications that are used during pregnancy and estimation of the risk of the spectrum of adverse pregnancy outcomes. However, depending on the recruitment strategy, assessment of miscarriages might not be feasible without introducing dedicated efforts to detect these.

### Conclusions

In this study, we found that first-trimester use of artemisinin derivatives was not associated with an increased risk of miscarriage or stillbirth compared to quinine. The data to date also indicate no difference in the prevalence of major anomalies between treatment groups in early pregnancy, although the numbers of major anomalies were small. Three-day ACT regimens are currently recommended to treat malaria in the second and third trimester. Expanding ACT recommendations to include the first trimester may outweigh the adverse outcomes of partially treated malaria due to poor adherence to 7-d oral quinine regimens in early pregnancy.

## Supporting information

S1 Checklist PRISMA-IPD checklist. (DOC)

S1 Fig Map of study sites included in the meta-analysis. (TIF)

S2 Fig Forest plot for unadjusted and adjusted hazard ratios for the association between confirmed artemisinin combination therapy treatment in the first trimester and miscarriage using a two-stage individual participant data approach. (TIF)

S3 Fig Forest plot for unadjusted and adjusted hazard ratios for the association between antimalarial treatment in the first trimester and stillbirth using a two-stage individual participant data approach. (TIF)

S4 Fig Forest plot showing the influence of removing one study at a time on the meta-analysis summary estimates for the effect of first-trimester antimalarial treatment on miscarriage. (TIF)

S1 Table Crude and adjusted hazard ratios for the association between different antimalarial treatment categories and miscarriage adjusting for HIV status, restricted to the individual participant data of the African sites. (DOCX)

S2 Table Number of documented cases of confirmed first-trimester artemisinin treatment. (DOCX)

S3 Table Summary of all congenital anomalies (minor and major) by Antiretroviral Pregnancy Registry Organ System Classification across treatment groups from the African sites, showing the difference after application of exclusion criteria as defined by the WHO Birth Defect Panel. (DOCX)

S4 Table Descriptive characteristics of pregnancies across exposure categories for the African sites contributing to the individual patient data meta-analysis for miscarriage. (DOCX)

S5 Table Descriptive characteristics of pregnancies across exposure categories for the African sites contributing to the individual patient data meta-analysis for congenital anomalies. (DOCX)

S6 Table Pooled prevalence of congenital malformations in the first trimester and in the embryo sensitive period for the study groups. (DOCX)

S1 Text Meta-analysis protocol. (DOCX)

S2 Text Characteristics of included studies. (DOCX)

S3 Text Inclusion and exclusion criteria from the Shoklo Malaria Research Unit and from the African site individual participant data. (DOCX)

S4 Text Statistical methods. (DOCX)

S5 Text Deviation from published data. (DOCX)

## Abbreviations

ACT: artemisinin combination therapy
aHR: adjusted hazard ratio
ASAP: Assessment of the Safety of Antimalarial Drug Use during Early Pregnancy
HR: hazard ratio
IPD: individual participant data
SMRU: Shoklo Malaria Research Unit
WHO: World Health Organization
