# Supplementary figures and images for: First-trimester artemisinin derivatives and quinine treatments and the risk of adverse pregnancy outcomes in Africa and Asia: A meta-analysis of observational studies
Source: PLoS Med. 2017 May 2;14(5):e1002290. doi: 10.1371/journal.pmed.1002290 (PMC5412992; doi:10.1371/journal.pmed.1002290)

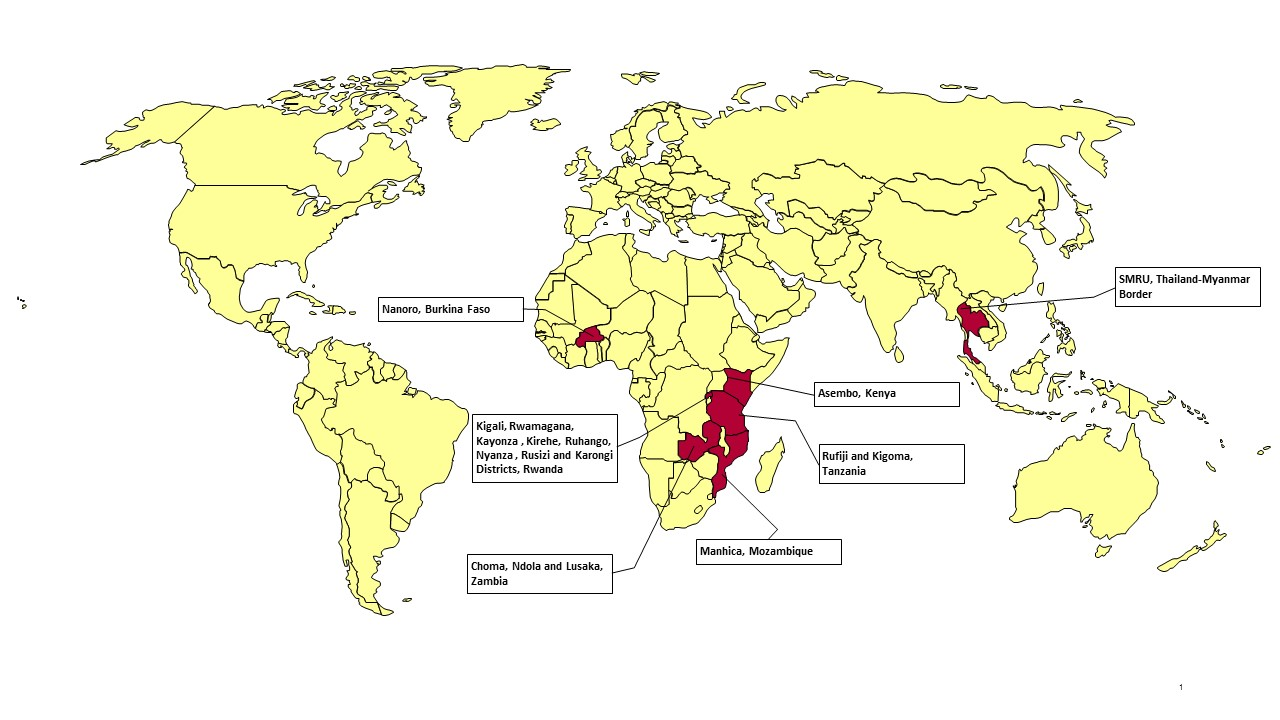

Supplement: S1 Fig — (TIF) [file pmed.1002290.s002.tif]

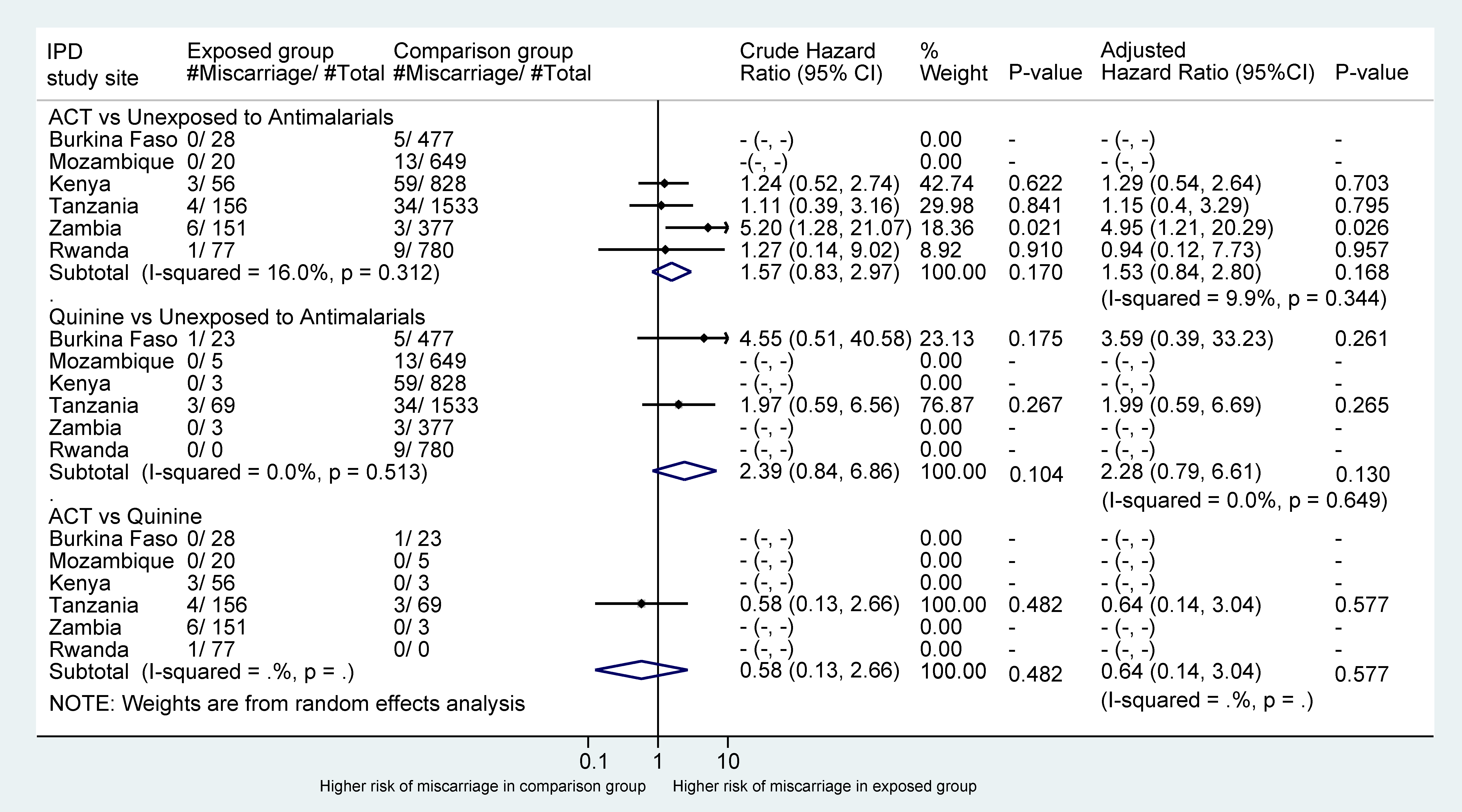

Supplement: S2 Fig — (TIF) [file pmed.1002290.s003.tif]

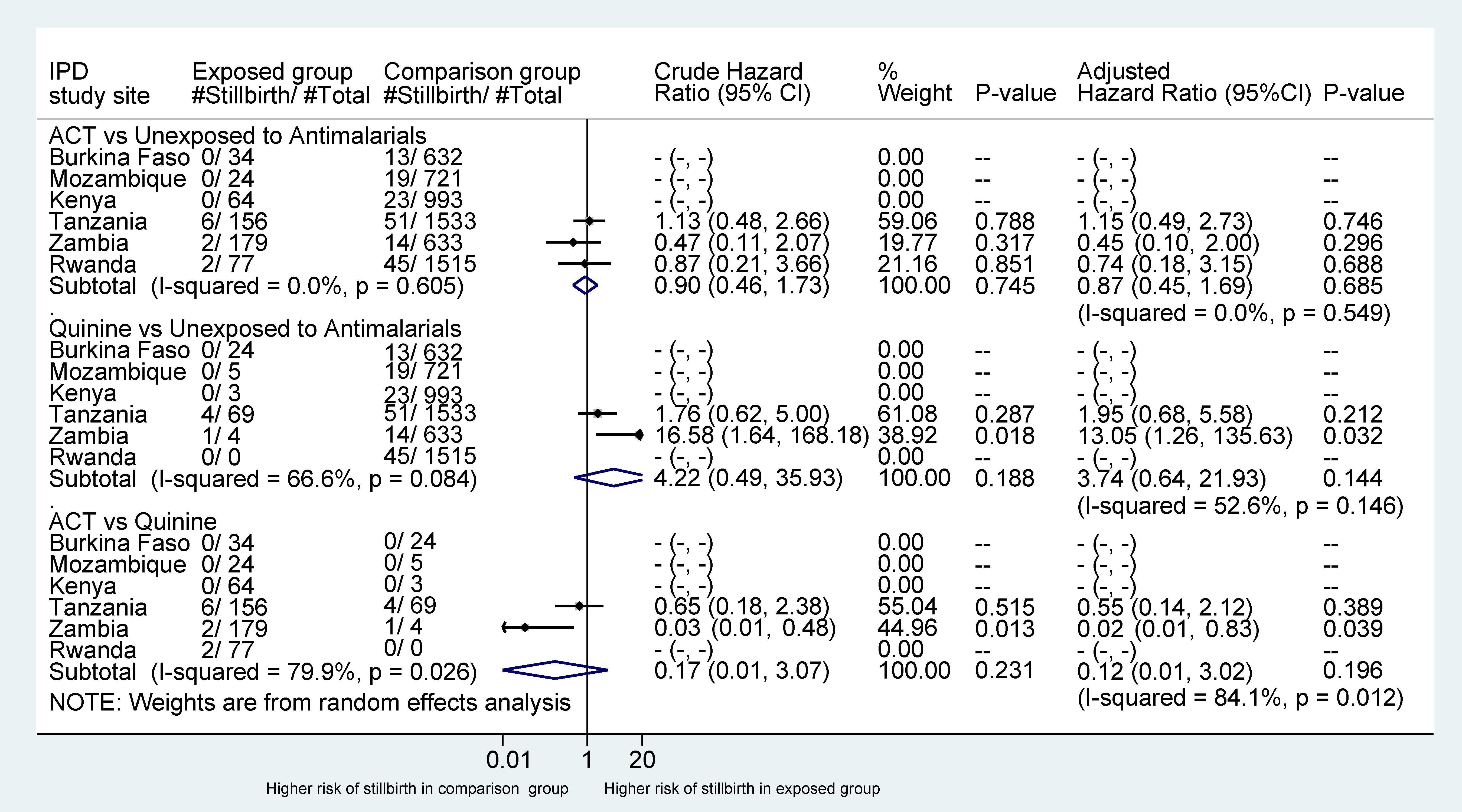

Supplement: S3 Fig — (TIF) [file pmed.1002290.s004.tif]

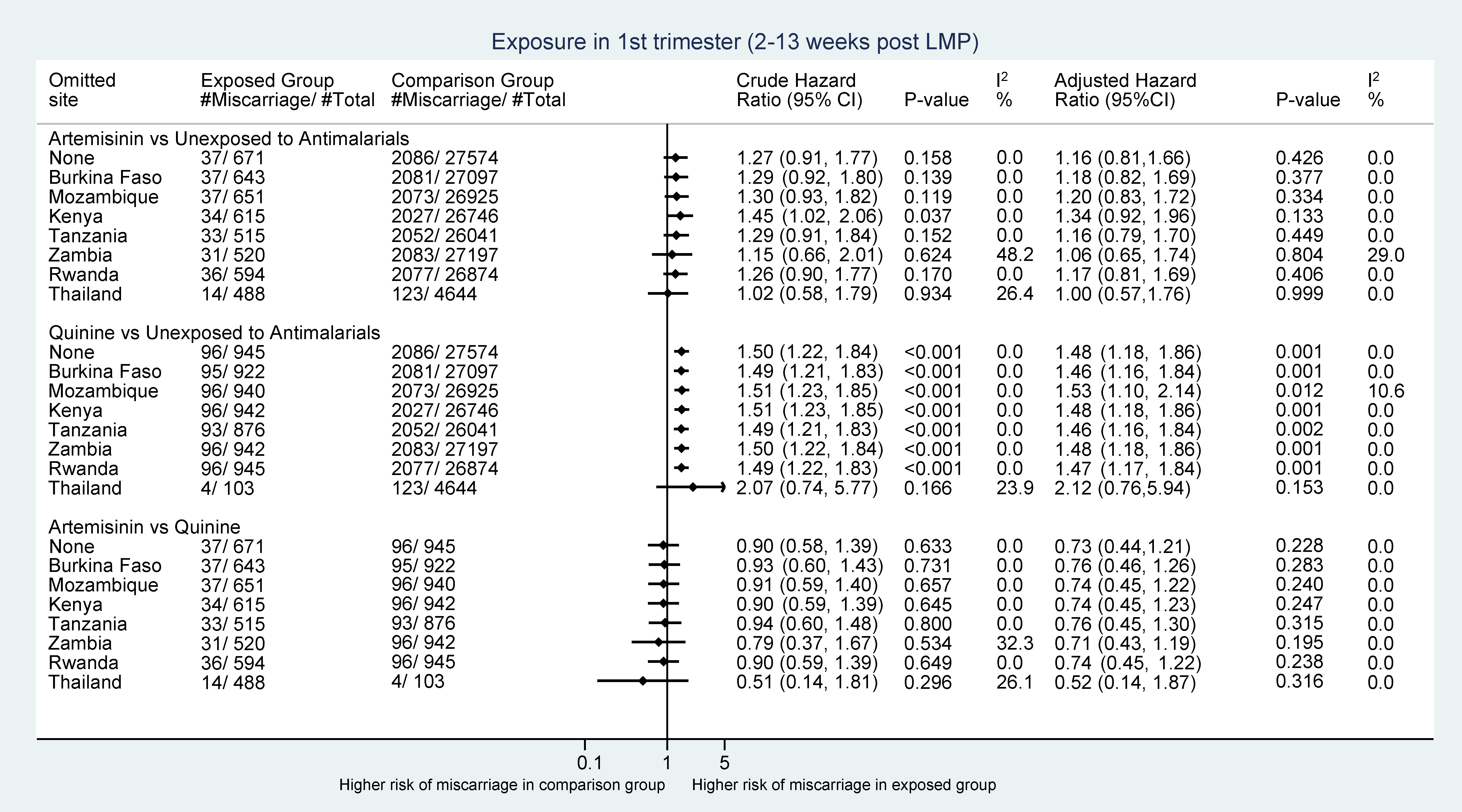

Supplement: S4 Fig — (TIF) [file pmed.1002290.s005.tif]
