# Supplementary material for: First-trimester artemisinin derivatives and quinine treatments and the risk of adverse pregnancy outcomes in Africa and Asia: A meta-analysis of observational studies
Source: PLoS Med. 2017 May 2;14(5):e1002290. doi: 10.1371/journal.pmed.1002290 (PMC5412992; doi:10.1371/journal.pmed.1002290)
Supplement: S1 Table — (DOCX) [file pmed.1002290.s006.docx]

| S1 Table. Crude and adjusted hazard ratios for the association between different antimalarial treatment categories and miscarriage adjusting for HIV status restricted to the IPD of African sites. | | | | | | | | |
| --- | --- | --- | --- | --- | --- | --- | --- | --- |
| **Antimalarial Treatment Categories** | **Crude Hazard ratio**  **(95%CI)** | **P-value** | **(A) Hazard ratio adjusted for age, gravidity and education (95%CI)** | **P-value** | **(B) Hazard ratio adjusted as in (A) and HIV using MI (95%CI)** | **P-value** | **(C) Hazard ratio adjusted as in (A) and HIV using categorical unknown(95%CI)** | **P-value** |
| ***ACT vs Unexposed to Antimalarials*** |  |  |  |  |  |  |  |  |
| Confirmed 1st trimester  (2-14 weeks post LMP) | 2.07 (0.74, 5.77) | 0.166 | 2.12 (0.76, 5.94) | 0.153 | 2.16 (0.78, 6.35) | 0.132 | 1.45 (0.81, 2.61) | 0.212 |
| Confirmed embryo sensitive  (6-12 weeks post LMP) | 1.03 (0.14, 7.56) | 0.978 | 1.04 (0.14, 7.65) | 0.971 | 1.04 (0.13, 7.42) | 0.942 | 1.47 (0.74, 2.90) | 0.270 |
| ***Quinine vs Unexposed to Antimalarials*** |  |  |  |  |  |  |  |  |
| Confirmed 1st trimester  (2-14 weeks post LMP) | 1.02 (0.58, 1.79) | 0.934 | 1.00 (0.57, 1.76) | 0.999 | 1.03 (0.52, 1.88) | 0.939 | 2.65 (0.94, 7.45) | 0.065 |
| Confirmed embryo sensitive  (6-12 weeks post LMP) | 1.08 (0.56, 2.08) | 0.811 | 1.08 (0.56, 2.09) | 0.808 | 0.95 (0.50, 1.90) | 0.881 | 1.26 (0.17, 9.32) | 0.820 |
| ***ACT vs Quinine*** |  |  |  |  |  |  |  |  |
| Confirmed 1st trimester  (2-14 weeks post LMP) | 0.51 (0.14, 1.81) | 0.296 | 0.52 (0.14, 1.87) | 0.316 | 0.57 (0.15, 2.15) | 0.335 | 0.55 (0.15, 2.02) | 0.370 |
| Confirmed embryo sensitive  (6-12 weeks post LMP) | 0.84 (0.33, 2.16) | 0.715 | 0.93 (0.36, 2.43) | 0.882 | 0.83 (0.31, 2.70) | 0.767 | 0.89 (0.33, 2.38) | 0.275 |
