# Supplementary material for: First-trimester artemisinin derivatives and quinine treatments and the risk of adverse pregnancy outcomes in Africa and Asia: A meta-analysis of observational studies
Source: PLoS Med. 2017 May 2;14(5):e1002290. doi: 10.1371/journal.pmed.1002290 (PMC5412992; doi:10.1371/journal.pmed.1002290)
Supplement: S2 Table — (DOCX) [file pmed.1002290.s007.docx]

| S2 Table. Number of documented confirmed 1st trimester artemisinins treatment | | | | | | | | | | |
| --- | --- | --- | --- | --- | --- | --- | --- | --- | --- | --- |
| **Author** | **Country** | **Publication Year** | **Number of confirmed first trimester treatment** | **AL** | **AS*** | **MAS** | **AS-SP** | **Art (IV/IM)** | **AQAS** | **DHA-PPQ** |
| **McGready [1]** | Thai-Myanmar Border | Updated and not yet published | 301 | 14 | 188 | 89 |  | 5^ |  | 5 |
| **Deen [2]** | Gambia | 2001 | 77 |  |  |  | 77 |  |  |  |
| **Adam [3]** | Sudan | 2009 | 62 | 3 |  |  | 11 | 48 |  |  |
| **Manyando [4]** | Zambia | 2010 | 156 | 156 |  |  |  |  |  |  |
| **Rulisa [5]** | Rwanda | 2012 | 96 | 96 |  |  |  |  |  |  |
| **Mosha [6]** | Tanzania | 2014 | 168 | 168 |  |  |  |  |  |  |
| **Poespoprodjo [7]** | Indonesia | 2014 | 18 |  |  |  |  | 10 |  | 13 |
| **Dellicour [8,9]** | Kenya | Not yet published | 85 | 85 |  |  |  |  |  |  |
| **Sevene [9]** | Mozambique | Not yet published | 21 | 21 |  |  |  |  |  |  |
| **Tinto [9]** | Burkina Faso | Not yet published | 41 | 1 |  |  |  |  | 40 |  |
| **Total** |  |  | **1025** | **544** | **188** | **89** | **88** | **58** | **40** | **18** |
| *AS for 7 days either as monotherapy n=147; or as a non-fixed combination including AS+Clindamycin n=36; AS + Doxycycline n=3; AS + atovaquone-proguanil n=2;  ^ Includes one women treated with Artemether IM for 6 days. | | | | | | | | | | |
| **Acronyms:** AL, artemether-lumefantrine; AQAS, amodiaquine-artesunate; AS, artesunate; AS-SP, artesunate-sulphadoxine-pyrimethamine; Art, artesunate; DHA-PPQ, dihydroartemisinin-piperaquine; IM, intramuscular; IV, intravenous; MAS: mefloquine-artesunate. | | | | | | | | | | |

**References**

1. McGready R, Lee SJ, Wiladphaingern J, Ashley EA, Rijken MJ, Boel M, et al. Adverse effects of falciparum and vivax malaria and the safety of antimalarial treatment in early pregnancy: a population-based study. Lancet Infect Dis. 2012;12(5):388-96. Epub 2011/12/16. doi: 10.1016/S1473-3099(11)70339-5

S1473-3099(11)70339-5 [pii]. PubMed PMID: 22169409; PubMed Central PMCID: PMC3346948.

2. Deen JL, von Seidlein L, Pinder M, Walraven GE, Greenwood BM. The safety of the combination artesunate and pyrimethamine-sulfadoxine given during pregnancy. Trans R Soc Trop Med Hyg. 2001;95(4):424-8. PubMed PMID: 11579889.

3. Adam I, Elhassan EM, Omer EM, Abdulla MA, Mahgoub HM, Adam GK. Safety of artemisinins during early pregnancy, assessed in 62 Sudanese women. Annals of tropical medicine and parasitology. 2009;103(3):205-10. Epub 2009/04/04. doi: 10.1179/136485909X398285. PubMed PMID: 19341535.

4. Manyando C, Mkandawire R, Puma L, Sinkala M, Mpabalwani E, Njunju E, et al. Safety of artemether-lumefantrine in pregnant women with malaria: results of a prospective cohort study in Zambia. Malar J. 2010;9:249. Epub 2010/09/03. doi: 10.1186/1475-2875-9-249

1475-2875-9-249 [pii]. PubMed PMID: 20809964; PubMed Central PMCID: PMC2944339.

5. Rulisa S, Kaligirwa N, Agaba S, Karema C, Mens PF, de Vries PJ. Pharmacovigilance of artemether-lumefantrine in pregnant women followed until delivery in Rwanda. Malar J. 2012;11:225. Epub 2012/07/10. doi: 10.1186/1475-2875-11-225

1475-2875-11-225 [pii]. PubMed PMID: 22770264; PubMed Central PMCID: PMC3407697.

6. Mosha D, Mazuguni F, Mrema S, Sevene E, Abdulla S, Genton B. Safety of artemether-lumefantrine exposure in first trimester of pregnancy: an observational cohort. Malar J. 2014;13(1):197. Epub 2014/06/03. doi: 10.1186/1475-2875-13-197

1475-2875-13-197 [pii]. PubMed PMID: 24884890; PubMed Central PMCID: PMC4040412.

7. Poespoprodjo JR, Fobia W, Kenangalem E, Lampah DA, Sugiarto P, Tjitra E, et al. Dihydroartemisinin-piperaquine treatment of multidrug resistant falciparum and vivax malaria in pregnancy. PLoS One. 2014;9(1):e84976. Epub 2014/01/28. doi: 10.1371/journal.pone.0084976

PONE-D-13-37045 [pii]. PubMed PMID: 24465458; PubMed Central PMCID: PMC3894943.

8. Dellicour S, Desai M, Aol G, Oneko M, Ouma P, Bigogo G, et al. Risks of miscarriage and inadvertent exposure to artemisinin derivatives in the first trimester of pregnancy: a prospective study in western Kenya. In preparation.

9. Tinto H, Sevene E, Dellicour S, Macete E, d’Alessandro U, ter Kuile FO, et al. Assessment of the Safety of Antimalarial Drug Use during Early Pregnancy: protocol for a multicenter prospective cohort study in Burkina Faso, Kenya and Mozambique. Reproductive Health. Submitted.
