# Supplementary material for: First-trimester artemisinin derivatives and quinine treatments and the risk of adverse pregnancy outcomes in Africa and Asia: A meta-analysis of observational studies
Source: PLoS Med. 2017 May 2;14(5):e1002290. doi: 10.1371/journal.pmed.1002290 (PMC5412992; doi:10.1371/journal.pmed.1002290)
Supplement: S3 Table — (DOCX) [file pmed.1002290.s008.docx]

| S3 Table. Summary of all congenital anomalies (minor+major) by APR Organ System Categories^29^ across first trimester treatment groups from the African sites before and after exclusion of anomalies not considered in the analysis | | | | | | | | |
| --- | --- | --- | --- | --- | --- | --- | --- | --- |
| **Group and Anomalies** | **All Congenital anomalies** | | | | **Congenital anomalies after exclusions** | | | |
|  | **No treatment**  **(N=4301)** | **ACT**  **(N=442)** | **Quinine**  **(N=100)** | **All Pregnancies**  **(N=4843)** | **No treatment**  **(N=4301)** | **ACT**  **(N=442)** | **Quinine**  **(N=100)** | **All Pregnancies**  **(N=4843)** |
| **Central Nervous System** |  |  |  |  |  |  |  |  |
| Anencephaly | 2 (0.04) | 0 (0.00) | 0 (0.00) | 2 (0.04) | 2 (0.05) | 0 (0.00) | 0 (0.00) | 2 (0.04) |
| Meningocele | 2 (0.04) | 0 (0.00) | 0 (0.00) | 2 (0.04) | 2 (0.05) | 0 (0.00) | 0 (0.00) | 2 (0.04) |
| **Face and Neck** |  |  |  |  |  |  |  |  |
| Abnormal insertion of ear | 1 (0.02) | 0 (0.00) | 0 (0.00) | 1 (0.02) | 1 (0.02) | 0 (0.00) | 0 (0.00) | 1 (0.02) |
| Auricular tag/pit | 43 (0.95) | 3 (0.67) | 0 (0.00) | 46 (0.90) | 6 (0.14) | 1 (0.23) | 0 (0.00) | 7 (0.14) |
| Accessory tragus | 1 (0.02) | 0 (0.00) | 1 (0.99) | 2 (0.04) | 0 (0.00) | 0 (0.00) | 0 (0.00) | 0 (0.00) |
| Hypertelorism | 1 (0.02) | 0 (0.00) | 0 (0.00) | 1 (0.02) | 1 (0.02) | 0 (0.00) | 0 (0.00) | 1 (0.02) |
| Chin depression | 2 (0.04) | 0 (0.00) | 0 (0.00) | 2 (0.04) | 0 (0.00) | 0 (0.00) | 0 (0.00) | 0 (0.00) |
| Unspecified anomaly of ear | 1 (0.02) | 0 (0.00) | 0 (0.00) | 1 (0.02) | 0 (0.00) | 0 (0.00) | 0 (0.00) | 0 (0.00) |
| Unspecified anomaly of eye | 3 (0.07) | 0 (0.00) | 0 (0.00) | 3 (0.06) | 1 (0.02) | 0 (0.00) | 0 (0.00) | 1 (0.02) |
| Unspecified anomaly of face | 1 (0.02) | 1 (0.22) | 0 (0.00) | 2 (0.04) | 0 (0.00) | 1 (0.23) | 0 (0.00) | 1 (0.02) |
| **Heart—Other Defects** |  |  |  |  |  |  |  |  |
| Unspecified anomaly | 4 (0.09) | 0 (0.00) | 0 (0.00) | 4 (0.08) | 4 (0.09) | 0 (0.00) | 0 (0.00) | 4 (0.08) |
| **Respiratory System** |  |  |  |  |  |  |  |  |
| Anomaly of larynx/trachea | 1 (0.02) | 0 (0.00) | 0 (0.00) | 1 (0.02) | 0 (0.00) | 0 (0.00) | 0 (0.00) | 0 (0.00) |
| **Lower Gastrointestinal System** |  |  |  |  |  |  |  |  |
| Absence/atresia of anus without fistula | 1 (0.02) | 0 (0.00) | 0 (0.00) | 1 (0.02) | 0 (0.00) | 0 (0.00) | 0 (0.00) | 0 (0.00) |
| **Female Genitalia** |  |  |  |  |  |  |  |  |
| Clitoromegaly | 3 (0.07) | 0 (0.00) | 0 (0.00) | 3 (0.06) | 3 (0.07) | 0 (0.00) | 0 (0.00) | 3 (0.06) |
| Imperforated urethral meatus | 1 (0.02) | 0 (0.00) | 0 (0.00) | 1 (0.02) | 0 (0.00) | 0 (0.00) | 0 (0.00) | 0 (0.00) |
| Small labia | 0 (0.00) | 1 (0.22) | 0 (0.00) | 1 (0.02) | 0 (0.00) | 1 (0.23) | 0 (0.00) | 1 (0.02) |
| **Male Genitalia** |  |  |  |  |  |  |  |  |
| Undescended testicles | 4 (0.09) | 0 (0.00) | 0 (0.00) | 4 (0.08) | 2 (0.05) | 0 (0.00) | 0 (0.00) | 2 (0.04) |
| Hypospadias NOS | 2 (0.04) | 0 (0.00) | 0 (0.00) | 2 (0.04) | 1 (0.02) | 0 (0.00) | 0 (0.00) | 1 (0.02) |
| **Group and Anomalies** | **All Congenital anomalies** | | | | **Congenital anomalies after exclusions** | | | |
|  | **No treatment (N=4301)** | **ACT**  **(N=442)** | **Quinine**  **(N=100)** | **All Pregnancies**  **(N=4843)** | **No treatment**  **(N=4301)** | **ACT**  **(N=441)** | **Quinine**  **(N=100)** | **All Pregnancies**  **(N=4843)** |
| Hernia inguinal | 1 (0.02) | 0 (0.00) | 0 (0.00) | 1 (0.02) | 0 (0.00) | 0 (0.00) | 0 (0.00) | 0 (0.00) |
| **Renal and Urinary System (RENAL)** |  |  |  |  |  |  |  |  |
| Imperforated urethral meatus | 1 (0.02) | 0 (0.00) | 0 (0.00) | 1 (0.02) | 1 (0.02) | 0 (0.00) | 0 (0.00) | 1 (0.02) |
| **Limb Reduction/Addition Defects** |  |  |  |  |  |  |  |  |
| Absence of hand/fingers | 1 (0.02) | 0 (0.00) | 0 (0.00) | 1 (0.02) | 1 (0.02) | 0 (0.00) | 0 (0.00) | 1 (0.02) |
| Polydactyly NOS—hand | 31 (0.68) | 3 (0.67) | 1 (0.99) | 35 (0.69) | 2 (0.05) | 1 (0.23) | 0 (0.00) | 3 (0.06) |
| Overlapping fingers | 1 (0.22) | 0 (0.00) | 0 (0.00) | 1 (0.02) | 1 (0.02) | 0 (0.00) | 0 (0.00) | 1 (0.02) |
| Rokaer-bottom feet | 1 (0.22) | 0 (0.00) | 0 (0.00) | 1 (0.02) | 1 (0.02) | 0 (0.00) | 0 (0.00) | 1 (0.02) |
| Syndactyly – toes | 2 (0.04) | 0 (0.00) | 0 (0.00) | 2 (0.04) | 2 (0.04) | 0 (0.00) | 0 (0.00) | 2 (0.04) |
| Other and unspecified polydactyly | 10 (0.22) | 1 (0.22) | 0 (0.00) | 11 (0.22) | 0 (0.00) | 0 (0.00) | 0 (0.00) | 0 (0.00) |
| Unspecified reduction defect of unspecified limb | 2 (0.04) | 0 (0.00) | 0 (0.00) | 2 (0.04) | 2 (0.05) | 0 (0.00) | 0 (0.00) | 2 (0.04) |
| **Other Musculoskeletal Defects** |  |  |  |  |  |  |  |  |
| Umbilical hernia | 61 (1.34) | 15 (3.33) | 0 (0.00) | 76 (1.49) | 10 (0.23) | 1 (0.23) | 0 (0.00) | 11 (0.23) |
| Congenital talipes/equinovarus | 13 (0.29) | 1 (0.22) | 0 (0.00) | 14 (0.27) | 3 (0.07) | 0 (0.00) | 0 (0.00) | 3 (0.06) |
| Sacral dimple | 39 (0.86) | 3 (0.67) | 0 (0.00) | 42 (0.82) | 3 (0.07) | 0 (0.00) | 0 (0.00) | 3 (0.06) |
| Omphalocele | 1 (0.02) | 0 (0.00) | 0 (0.00) | 1 (0.02) | 1 (0.02) | 0 (0.00) | 0 (0.00) | 1 (0.02) |
| **Skin and Skin Derivatives** |  |  |  |  |  |  |  |  |
| Accessory/ectopic/supernumerary nipple | 2 (0.04) | 1 (0.22) | 0 (0.00) | 3 (0.06) | 0 (0.00) | 1 (0.23) | 0 (0.00) | 1 (0.02) |
| Retracted nipple | 3 (0.07) | 1 (0.22) | 0 (0.00) | 4 (0.08) | 0 (0.00) | 0 (0.00) | 0 (0.00) | 0 (0.00) |
| Inverted nipple | 7 (0.15) | 1 (0.22) | 0 (0.00) | 8 (0.16) | 2 (0.05) | 0 (0.00) | 0 (0.00) | 2 (0.04) |
| Skin tag | 3 (0.07) | 1 (0.22) | 0 (0.00) | 4 (0.08) | 1 (0.02) | 0 (0.00) | 0 (0.00) | 1 (0.02) |
| Hyperpigmentation | 2 (0.04) | 0 (0.00) | 0 (0.00) | 2 (0.04) | 0 (0.00) | 0 (0.00) | 0 (0.00) | 0 (0.00) |
| Dermal Cyst | 1 (0.02) | 0 (0.00) | 0 (0.00) | 1 (0.02) | 0 (0.00) | 0 (0.00) | 0 (0.00) | 0 (0.00) |
| Lanugo | 0 (0.00) | 1 (0.22) | 0 (0.00) | 1 (0.02) | 0 (0.00) | 1 (0.23) | 0 (0.00) | 1 (0.02) |
| Unspecified anomaly of skin | 1 (0.02) | 0 (0.00) | 0 (0.00) | 1 (0.02) | 0 (0.00) | 0 (0.00) | 0 (0.00) | 0 (0.00) |
| **Total anomalies** | **256 (5.95)** | **33 (7.47)** | **2 (2.00)** | **291 (6.01)** | **53 (1.23)** | **7 (1.58)** | **0 (0.00)** | **60 (1.24)** |
| **Births with anomalies** | **225 (5.23)** | **26 (5.88)** | **2 (2.00)** | **253 (5.22)** | **29 (0.67)** | **3 (0.68)** | **0 (0.00)** | **32 (0.66)** |
| **Births with major anomalies s** | **30 (0.70)** | **5 (1.13)** | **0 (0.00)** | **35 (0.72)** | **18 (0.42)** | **0 (0.00)** | **0 (0.00)** | **18 (0.37)** |
