# Supplementary material for: First-trimester artemisinin derivatives and quinine treatments and the risk of adverse pregnancy outcomes in Africa and Asia: A meta-analysis of observational studies
Source: PLoS Med. 2017 May 2;14(5):e1002290. doi: 10.1371/journal.pmed.1002290 (PMC5412992; doi:10.1371/journal.pmed.1002290)
Supplement: S4 Table — (DOCX) [file pmed.1002290.s009.docx]

| S4 Table. Descriptive characteristics of pregnancies across exposure categories for the African sites contributing to the individual patient data meta-analysis for miscarriage | | | | | | | | |
| --- | --- | --- | --- | --- | --- | --- | --- | --- |
|  | **All Pregnancies in miscarriage analysis** | | **No Antimalarial use 1^st^ trimester** | | **Confirmed ACT use 1^st^ trimester** | | **Confirmed Quinine use 1^st^ trimester** | |
|  | **N=5,235** | | **N=4,644** | | **N=488** | | **N=103** | |
|  | N | (%) | N | (%) | n | (%) | n | (%) |
| **Age (years)** |  |  |  |  |  |  |  |  |
| Mean (SD) | 25.8 | (6.5) | 25.9 | (6.5) | 25.8 | (6.1) | 25.3 | (5.9) |
| <20 | 946 | (18.1) | 854 | (18.4) | 72 | (14.8) | 20 | (19.4) |
| 20-24 | 1547 | (29.6) | 1356 | (29.2) | 165 | (33.8) | 26 | (25.2) |
| 25-29 | 1263 | (24.1) | 1114 | (24.0) | 116 | (23.8) | 33 | (32.0) |
| 30+ | 1479 | (28.3) | 1320 | (28.4) | 135 | (27.7) | 24 | (23.3) |
| **Gravidity** |  |  |  |  |  |  |  |  |
| Primigravida | 1375 | (26.3) | 1193 | (25.7) | 148 | (30.3) | 34 | (33.0) |
| 1–3 pregnancies | 2558 | (48.9) | 2273 | (48.9) | 235 | (48.2) | 50 | (48.5) |
| 4+ pregnancies | 1205 | (23.0) | 1084 | (23.3) | 104 | (21.3) | 17 | (16.5) |
| Missing | 97 |  | 94 |  | 1 |  | 2 |  |
| **Marital status** |  |  |  |  |  |  |  |  |
| Single | 753 | (14.4) | 696 | (15.0) | 48 | (9.8) | 9 | (8.7) |
| Married or living together | 3006 | (57.4) | 2713 | (58.4) | 203 | (41.6) | 90 | (87.4) |
| Missing | 1476 |  | 1235 |  | 237 |  | 4 |  |
| **Education** |  |  |  |  |  |  |  |  |
| Primary not completed | 1337 | (25.5) | 1230 | (26.5) | 81 | (16.6) | 26 | (25.2) |
| Primary completed | 2200 | (42.0) | 1925 | (41.5) | 219 | (44.9) | 56 | (54.4) |
| Secondary completed | 771 | (14.7) | 645 | (13.9) | 106 | (21.7) | 20 | (19.4) |
| Missing | 927 |  | 844 |  | 82 |  | 1 |  |
| **HIV status** |  |  |  |  |  |  |  |  |
| Negative | 4423 | (84.5) | 3894 | (83.9) | 432 | (88.5) | 97 | (94.1) |
| Positive | 478 | (9.1) | 444 | (9.6) | 32 | (6.6) | 2 | (1.9) |
| Missing | 334 |  | 306 |  | 24 |  | 4 |  |
| **Gestational age in weeks at enrollment** |  |  |  |  |  |  |  |  |
| Mean (SD) | 17.4 | (6.3) | 17.8 | (6.2) | 13.8 | (5.9) | 14.2 | (4.3) |
| Median (IQR) | 17 | (13–22) | 18 | (14–23) | 13 | (9–17) | 14 | (12–16) |
| **Duration of follow-up in weeks** |  |  |  |  |  |  |  |  |
| Mean (SD) | 11.6 | (6.3) | 11.2 | (6.2) | 15.2 | (5.6) | 14.8 | (4.3) |
| Median (IQR) | 12 | (7-16) | 11 | (6-15) | 16 | (11-19) | 15 | (12-17) |
