# Supplementary material for: First-trimester artemisinin derivatives and quinine treatments and the risk of adverse pregnancy outcomes in Africa and Asia: A meta-analysis of observational studies
Source: PLoS Med. 2017 May 2;14(5):e1002290. doi: 10.1371/journal.pmed.1002290 (PMC5412992; doi:10.1371/journal.pmed.1002290)
Supplement: S5 Table — (DOCX) [file pmed.1002290.s010.docx]

| S5 Table | Descriptive characteristics of pregnancies across exposure categories for the African sites contributing to the congenital anomalies data analysis. | | | | | | | | |
| --- | --- | --- | --- | --- | --- | --- | --- | --- | --- |
|  | | **All Pregnancies**  **N=4,843** | | **No Antimalarial use 1^st^ trimester N=4,301** | | **Confirmed ACT use 1^st^ trimester N=442** | | **Confirmed Quinine use 1^st^ trimester N=100** | |
|  | | N | (%) | N | (%) | n | (%) | n | (%) |
| **Age (years)** | |  |  |  |  |  |  |  |  |
| Mean (SD) | | 25.7 | (6.5) | 25.7 | (6.6) | 25.5 | (6.1) | 25.4 | (5.7) |
| <20 | | 963 | (28.6) | 873 | (20.3) | 71 | (16.1) | 19 | (19.0) |
| 20-24 | | 1385 | (24.3) | 1206 | (28.0) | 153 | (34.6) | 26 | (26.0) |
| 25-29 | | 1175 | (24.3) | 1042 | (24.2) | 100 | (22.6) | 33 | (33.0) |
| 30+ | | 1318 | (27.2) | 1178 | (27.4) | 118 | (26.7) | 22 | (22.0) |
| **Gravidity** | |  |  |  |  |  |  |  |  |
| Primigravida | | 1241 | (25.6) | 1083 | (25.1) | 124 | (28.1) | 34 | (34.0) |
| 1–3 pregnancies | | 2433 | (50.2) | 2165 | (50.3) | 221 | (50.0) | 47 | (47.0) |
| 4+ pregnancies | | 1158 | (23.9) | 1044 | (24.3) | 97 | (22.0) | 17 | (17.0) |
| Missing | | 11 |  | 9 |  |  |  | 2 |  |
| **Marital status** | |  |  |  |  |  |  |  |  |
| Single | | 771 | (15.9) | 714 | (16.6) | 48 | (10.9) | 9 | (9.0) |
| Married or living together | | 3226 | (66.6) | 2927 | (68.1) | 213 | (48.2) | 86 | (86.0) |
| Missing | | 846 |  | 660 |  | 181 |  | 5 |  |
| **Education** | |  |  |  |  |  |  |  |  |
| Primary not completed | | 890 | (18.4) | 822 | (19.1) | 61 | (13.8) | 7 | (7.0) |
| Primary completed | | 3017 | (62.3) | 2687 | (62.5) | 258 | (58.4) | 72 | (72.0) |
| Secondary completed | | 915 | (18.9) | 776 | (18.0) | 119 | (26.9) | 20 | (20.0) |
| Missing | | 21 |  | 16 |  | 4 |  | 1 |  |
| **HIV status** | |  |  |  |  |  |  |  |  |
| Negative | | 4112 | (84.9) | 3636 | (84.5) | 383 | (86.7) | 93 | (93.0) |
| Positive | | 504 | (10.4) | 467 | (10.9) | 35 | (7.9) | 2 | (2.0) |
| Missing | | 227 |  | 198 |  | 24 |  | 5 |  |
| **Gestational age in weeks at enrollment** | |  |  |  |  |  |  |  |  |
| Mean (SD) | | 19.3 | (7.8) | 19.6 | (7.8) | 17.6 | (7.7) | 14.6 | (5.1) |
| Median (IQR) | | 18 | (14–24) | 18 | (14–25) | 16 | (12–22) | 14 | (12–17) |
| **Duration of follow-up in weeks** | |  |  |  |  |  |  |  |  |
| Mean (SD) | | 18.8 | (7.8) | 18.5 | (7.8) | 20.4 | (7.9) | 22.5 | (5.7) |
| Median (IQR) | | 19 | (13–24) | 19 | (13–23) | 21 | (16–26) | 23 | (20–26) |
