# Supplementary material for: First-trimester artemisinin derivatives and quinine treatments and the risk of adverse pregnancy outcomes in Africa and Asia: A meta-analysis of observational studies
Source: PLoS Med. 2017 May 2;14(5):e1002290. doi: 10.1371/journal.pmed.1002290 (PMC5412992; doi:10.1371/journal.pmed.1002290)
Supplement: S6 Table — (DOCX) [file pmed.1002290.s011.docx]

S6 Table : Pooled prevalence of congenital anomalies in first trimester and in embryo-sensitive period between the study groups.

| **First trimester** |  |  |  |  | **Embryo-sensitive period** | | |  |  |
| --- | --- | --- | --- | --- | --- | --- | --- | --- | --- |
| **Site** | **N** | **cases** | **Prevalence per 1000** | | **Site** | **N** | **cases** | **Prevalence per 1000** | |
|  |  |  | **Estimate** | **95CI** |  |  |  | **Estimate** | **95CI** |
| **Artemisinins** |  |  |  |  | **Artemisinins** |  |  |  |  |
| Burkina Faso | 34 | 1 | 29 | 1 - 153 | Burkina Faso | 23 | 1 | 43 | 0 - 148 |
| Mozambique | 24 | 0 | 0 | 0 - 142 | Mozambique | 24 | 0 | 0 | 0 - 142 |
| Kenya | 59 | 1 | 17 | 0 - 91 | Kenya | 36 | 0 | 0 | 0 - 97 |
| Tanzania | 152 | 0 | 0 | 0 - 24 | Tanzania | 135 | 0 | 0 | 0 - 27 |
| Zambia | 173 | 1 | 6 | 0 - 32 | Zambia | 115 | 1 | 9 | 0 - 32 |
| Thailand | 109 | 2 | 18 | 2 - 65 | Thailand | 54 | 2 | 37 | 2 - 65 |
| *African pooled* |  |  | 0 | 0 - 37 | *African pooled* |  |  | 16 | 4 - 58 |
| *All pooled* |  |  | 15 | 6 - 35 | *All pooled* |  |  | 24 | 9 - 61 |
| **Quinine** |  |  |  |  | **Quinine** |  |  |  |  |
| Burkina Faso | 23 | 0 | 0 | 0 - 148 | Burkina Faso | 13 | 0 | 0 | 0 - 247 |
| Mozambique | 4 | 0 | 0 | 0 - 602 | Mozambique | 4 | 0 | 0 | 0 - 602 |
| Kenya | 3 | 0 | 0 | 0 - 708 | Kenya | 1 | 0 | 0 | 0 - 975 |
| Tanzania | 66 | 0 | 0 | 0 - 54 | Tanzania | 31 | 0 | 0 | 0 - 112 |
| Zambia | 4 | 0 | 0 | 0 - 602 | Zambia | 3 | 0 | 0 | 0 - 708 |
| Thailand | 641 | 8 | 12 | 2 - 65 | Thailand | 517 | 8 | 15 | 7 - 30 |
| *African pooled* |  |  | 5 | 0 - 212 | *African pooled* |  |  | 9 | 0 - 316 |
| *All pooled* |  |  | 12 | 6 - 24 | *All pooled* |  |  | 15 | 8 - 30 |
| **Non exposed** |  |  |  |  | **Non exposed** |  |  |  |  |
| Burkina Faso | 627 | 4 | 6 | 2 - 16 | Burkina Faso | 627 | 4 | 6 | 2 - 16 |
| Mozambique | 688 | 3 | 4 | 1 - 13 | Mozambique | 688 | 3 | 4 | 1 - 13 |
| Kenya | 857 | 18 | 21 | 13 - 33 | Kenya | 857 | 18 | 21 | 13 - 33 |
| Tanzania | 1499 | 3 | 2 | 0 - 6 | Tanzania | 1499 | 3 | 2 | 0 - 6 |
| Zambia | 630 | 1 | 2 | 0 - 9 | Zambia | 630 | 1 | 2 | 0 - 9 |
| Thailand | 18803 | 158 | 8 | 7 - 10 | Thailand | 18803 | 158 | 8 | 7 - 10 |
| *African pooled* |  |  | 5 | 2 - 15 | *African pooled* |  |  | 5 | 2 - 15 |
| *All pooled* |  |  | 7 | 4 - 12 | *All pooled* |  |  | 7 | 4 - 12 |
|  |  |  |  |  |  |  |  |  |  |
| **Total** |  |  |  |  | **Total** |  |  |  |  |
| Burkina Faso | 684 | 5 | 7 | 2 - 15 | Burkina Faso | 663 | 5 | 8 | 2 - 15 |
| Mozambique | 716 | 3 | 4 | 1 - 12 | Mozambique | 716 | 3 | 4 | 1 - 12 |
| Kenya | 919 | 19 | 21 | 4 - 17 | Kenya | 894 | 18 | 20 | 4 - 18 |
| Tanzania | 1717 | 3 | 2 | 0 - 5 | Tanzania | 1665 | 3 | 2 | 0 - 5 |
| Zambia | 807 | 2 | 2 | 0 - 5 | Zambia | 748 | 2 | 3 | 0 - 5 |
| Thailand | 19553 | 168 | 9 | 7 - 10 | Thailand | 19374 | 168 | 9 | 7 - 10 |
| *African pooled* |  |  | 5 | 7 - 15 | *African pooled* |  |  | 5 | 2 - 15 |
| *All pooled* |  |  | 7 | 4 - 12 | *All pooled* |  |  | 7 | 4 - 12 |
|  |  |  |  |  |  |  |  |  |  |
