# Supplementary material for: First-trimester artemisinin derivatives and quinine treatments and the risk of adverse pregnancy outcomes in Africa and Asia: A meta-analysis of observational studies
Source: PLoS Med. 2017 May 2;14(5):e1002290. doi: 10.1371/journal.pmed.1002290 (PMC5412992; doi:10.1371/journal.pmed.1002290)
Supplement: S1 Text — (DOCX) [file pmed.1002290.s012.docx]

| S1 Text | Meta-analysis protocol |
| --- | --- |

# **Pooled Analysis for the Assessment of the Safety of Exposure to Artemisinin Derivatives in the first trimester of Pregnancy**

## Investigators:

Liverpool School of Tropical Medicine, UK: Stephanie Dellicour and Feiko ter Kuile

Manhica Health Research Centre, Mozambique: Esperanca Sevene and Orvalho Augusto

IRSS/Centre Muraz, Burkina Faso: Halidou Tinto

University of Illinois, Department of Pharmacy Systems, Outcomes and Policy, USA: Greg Calip

University of Washington Department of Global Health, USA: Andy Stergachis

## Background

Every year, around 125 million women living in malaria endemic countries worldwide become pregnant.[1] Pregnant women need safe and effective antimalarial treatments and prevention measures to avoid substantial adverse consequences for the mother, her developing fetus, newborn, and infant.[2] A retrospective study in Thailand, reported the deleterious effect of even a single episode of malaria in the first trimester and its association with a 3-4 fold increase in the risk of miscarriage, emphasising the need for safe and efficacious drugs in this critical period.[3] This is further supported by a recent modelling study reporting that up to two thirds of placental infections could occur by the end of the first trimester without prevention.[4] Antimalarials recommended for pregnant patients must be safe for the mother and her unborn baby. There is insufficient information on the safety of most antimalarials in pregnancy, particularly for exposure in the first trimester. ACTs are among the most effective and rapidly acting antimalarials to date, providing life-saving benefits to children, adults, and pregnant women in the 2^nd^ and 3^rd^ trimesters. The current recommendation for pregnant women in their first trimester with uncomplicated malaria is 7 days treatment with quinine+clyndamicin.[5] WHO currently only recommend the use of ACTs in the first trimester if there is no alternative and the life of the woman is in danger.[5] This is based on pre-clinical animal data showing teratogenicity and limited data on human exposures. Animal reprotoxicology studies showed that artemisinin derivatives have embryotoxic effects in all species studied (i.e. rat, rabbit and monkey) at low dose ranges.[6, 7] Pre-clinical studies showed that the mechanism of embryotoxicity was through insult to immature red blood cells (primitive erythroblasts) causing severe anaemia in the embryo and leading to either embryolethality or malformations, skeletal (shortened or bent long bones and scapulae, misshapen ribs, cleft sternebrae and incompletely ossified pelvic bones) and cardiovascular (ventricular septal and vessel defects).[6, 8] These studies also predicted that the main window for insult to the fetus will occur early in pregnancy (between 4 and 10 weeks post conception).[6] This is the period when the nucleated, metabolically active primitive erythroblasts predominate in the blood. Information regarding risks associated with the use of antimalarials in pregnancy in humans is sparse. Although the limited data on human exposures is reassuring for first trimester exposures, further information is required.[9-11]

WHO published a report in 2007 reviewing all available data on the reproductive toxicology (pre-clinical) and human clinical data of artemisinin compounds in pregnancy. The report concluded that there was insufficient evidence at the time to warrant a change in WHO policy recommendations on the use of artemisinin-based products for the treatment of malaria in early pregnancy.[12] Since then several studies looking at the safety of inadvertent exposures to ACT in the first trimester have been concluded and present the opportunity to revaluate the evidence available to date. However the published studies have used varying methodological and analytical approaches to report on the safety of artemisinin in pregnancy and synthesis of the evidence would require standardisation across studies. This could be achieved through pooled analysis of individual patient data (IPD) and meta-analysis of aggregated data following standardisation of analytical strategies, exposure and endpoint definitions.

## Aims and objectives

The aim of this collaborative pooled analysis is to determine the level of risk that can be excluded for the association between artemisinin derivative first trimester exposures and adverse pregnancy outcomes.

The specific objectives are:

1. To compare the risk of miscarriage, stillbirths and congenital malformations for women exposed to artemisinin in the first trimester to women exposed to quinine in the same period accounting for potential confounding factors and effect modifier and time of entry in the study.
2. To carry out sensitivity analysis by repeating the analyses above but
   1. Comparing artemisinin exposed pregnancies to pregnancies unexposed to antimalarials
   2. Comparing quinine exposed pregnancies to pregnancies unexposed to antimalarials
   3. Assessing artemisinin exposure in the suggested artemisinin embryo-sensitive period.

## Methods

### Study selection criteria

#### Types of studies

Prospective cohort studies with internal comparison groups

#### Types of participants

Pregnant women enrolled before pregnancy outcome was known (i.e. prospective follow up of pregnancy)

#### Interventions

Artemisinin derivatives or quinine used in the first trimester of pregnancy for treatment of malaria, exposure ascertainment needs to be confirmed through multiple data sources

#### Exposure group definitions

Exposures will be considered confirmed if information could be verified across at least 2 data sources (for example self-reported by the woman and confirmed from the clinic registers) or treatment were directly observed. Pregnancies treated with both artemisinins and quinine or who had more than one of antimalarial treatment course in the first trimester will be excluded from the IPD analysis.

- Artemisinins: The exposure of interest is artemisinin used in the first trimester of pregnancy (<=13 weeks from the date of the last menstrual period [LMP] inclusive) and artemisinin used in the suspected embryo sensitive period (6-12 weeks post LMP inclusive).
- Quinine: is the recommended first line treatment for uncomplicated malaria in the first trimester and is considered safe. Therefore, oral quinine treatment will be the primary comparator.
- Unexposed: The group with no antimalarial treatment will include pregnancies that were confirmed to not have been treated with antimalarials between 2-18 weeks gestation, i.e. the first trimester plus a four week lag-period, according to any of the data sources for the IPD analysis.

#### Outcomes

Outcomes of interests encompassed the following adverse pregnancy outcomes:

- Miscarriage: defined as a confirmed pregnancy that fails to progress, resulting in death and expulsion of the embryo or unviable fetus (< 28 weeks). Miscarriages will be analysed as a dichotomous variable either present or absent.
- Stillbirth: defined as a confirmed pregnancy lasting until 28 weeks of pregnancy, that results in the birth of a stillborn infant.
- Major congenital malformation: defined as any structural abnormality with surgical, medical or cosmetic importance that is present at birth. Cases with two or more minor congenital anomalies will also be considered as major. The birth defect organ system classification developed by the Antiretroviral Pregnancy Registry[13], which groups defects of presumed common embryologic pathogenesis, will be applied. Major congenital malformations will be dichotomized as any major malformation compared to none.

#### Search methods for identification of studies

A prospective IPD analysis is planned for three sites under a single multi-centre study protocol: the Assessment of the Safety of Antimalarial drug use during Pregnancy study (ASAP).[14] Additional eligible studies will be identified through a systematic literature search. The electronic search will be carried out through Medline, Embase and the Malaria in Pregnancy Consortium Library using keywords and MeSH terms with no restriction for time of publication or language. The search terms for Medline consisted of: (Pregnant women OR pregnan* AND malaria) AND (Artemisinin* OR “Artemisinin Combination Therapy” OR ACT OR artemether OR artesunate OR dihydroartemisinin OR treatment) AND (Pregnancy complication [mh] OR safety OR “serious adverse event” OR miscarriage OR stillbirth OR “pregnancy loss” OR “spontaneous abortion” OR “birth defect” OR congenital abnormalities OR “congenital malformations” OR “congenital anomalies”) AND cohort study [mh] OR prospective [tw]. We will also search 'gray literature' databases, conference abstracts and manually reviewed reference lists of selected publications. Unpublished information will be solicited from individual researchers and organizations working in the field.

#### Selection of studies

All records identified through the search described above will be reviewed and duplicate records removed. Abstracts will be screened and papers that do not meet the inclusion criteria in relation to study design, study participants or intervention will be excluded. Of the remainder, full text articles will be assessed for eligibility against the eligibility criteria screening by two independent reviewers. Any discrepancy will be discussed until an agreement has been reached with consultation of a third party if needed.

#### Quality assessment

The Newcastle Ottawa Scale for assessing bias in cohort studies will be used[15] with assessment of the selection of the exposed and unexposed groups, the comparability of the groups and the ascertainment of the outcomes of interest including attrition. Studies will be classified as low risk of bias (7-9 stars, bias is unlikely to alter the results seriously), moderate risk (4-6 stars, a risk of bias that raises some doubt about the results), and high risk of bias (0-3 stars, bias may alter the results seriously).

### Data collection processes

All potential collaborators will be contacted to gage interest and to assess feasibility of carrying out IPD pooled analysis. A data sharing agreement will be signed with all collaborators agreeing to participate. A list of core data elements to be requested from each study is provided in table A. If sharing IPD is not possible, investigators will be invited to share aggregated data and effect estimates following the standardised data analysis approach used for the IPD.

Data submitted by investigators for the pooled analysis will be checked for internal consistency, outliers and missing values before pooling across studies. All collaborators will be expected to clarify any data query as well as review and input on the report with consultation with colleagues from the studies they represent. In the case of missing data on key covariates and confounders, we will perform quality checks with the individual study sites to attempt to ascertain the missing values. In sensitivity analysis, we will assess the impact of applying multiple imputation methods for key confounding variables and determine how to handle the missing data and the strength of assumptions made.

Standardisation of variables will be ensured at the central level such as the definition of the main exposure of interest including timing (e.g. first trimester will be defined as 2 weeks 0 day from first day of last menstrual period to 14weeks 0 day) as well as outcome definition (e.g. miscarriage will be defined as pregnancy loss before 28 weeks of gestation and stillbirths at or above 28 weeks).

### Confidentiality and data handling

All data will need to be de-identified and handled according to Good Clinical Practice. All raw data will be transferred securely (via secure email, or secure electronic transfer) and stored on a secure server in a password protected database. The data supplied by each collaborator will be used only for the purposes stated in the analysis plan.

### Data Analysis

We will use a combination of IPD and meta-analysis of aggregated data if needed. We will complete IPD of data from the sites that agree to share individual level data. We will restrict the analysis for participants recruited prospectively during the index pregnancy (and exclude participants recruited after pregnancy outcome).

*IPD pooled analysis:* To estimate the relation between artemisinin exposures and risk of adverse pregnancy outcomes, we will use Cox proportional hazards models, accounting for time under observation and different gestational age at enrolment, to calculate hazard ratios (HR) and 95% confidence intervals (CI) with adjustment for potential confounders. Analyses of fetal loss should account for left truncation [16] as the risk decreases rapidly with increasing gestation. Accounting for left truncation is essential to avoid significant bias when gestational age at exposure and enrolment vary between comparison groups [17] (e.g. more inadvertent treatments to artemisinins very early in the first trimester when women do not yet realise or report they may be pregnant and the risk of miscarriage is highest, versus more exposure to quinine later in the first trimester when the pregnancy is evident and the risk of miscarriage much lower). Exposure will be treated as time-dependant so that participants are considered exposed only from the time they received artemisinin or quinine treatment rather than from the time of enrolment in the study. The unexposed group will consist of a combination of pregnancies never exposed and of pregnancies that were eventually exposed, but will contribute ‘unexposed pregnancy-weeks’ to the unexposed category until the day of exposure, after which they will switch to the exposed category. For example if a participant was enrolled in the study at gestational week 8 but received treatment at gestational week 11, she will be considered as exposed from week 11 and contributed person-time for the unexposed between weeks 8-11. Careful examination of the validity of the models and testing of the proportional hazards assumption in Cox regression will be performed. Firstly, we will conduct a pooled analysis in a single harmonized dataset, with HRs and 95% CIs for artemisinin exposures computed with Cox proportional hazards models with adjustment for study site and potential confounders (1-stage IPD). We will evaluate potential confounding of key covariates in our analysis using descriptive statistics and in the model-building phase.

Secondly, we will also compute HRs and 95% CIs within each study and conduct a meta-analysis of study-specific findings using both random- and fixed-effects models (2-stage IPD). Heterogeneity in the meta-analysis will be assessed using the using *Q* statistic and I^2^ interpreted as follows: 0-40% heterogeneity may not be important; 30-60% moderate heterogeneity; 50-90% high heterogeneity and >75% considerable heterogeneity.

*Aggregated data meta-analysis:* We will then use the effect estimates of the IPD analyses for miscarriage, stillbirth and congenital anomalies and combine these with similar effect estimates from aggregated data using random effects meta-analyses from any sites wishing to share aggregated data instead of IPD. Heterogeneity in the meta-analysis will be assessed using the I^2^ statistic.

We plan to conduct a sensitivity analysis by restricting exposures to the artemisinin embryo-sensitive period (4-10 weeks post conception). We will also conduct a sensitivity analysis using participants unexposed to antimalarials as a comparison. We will assess the influence of individual studies by repeating the analysis removing one study at a time.

### Authorship and publication policy

The primary purpose of this collaborative pooled analysis is to prepare a report for the WHO ERG meeting on the latest evidence relating to the risk of adverse pregnancy outcome associated with artemisinin exposures in the first trimester. MiPc will lead the collation of data, the analysis and drafting of the report. All collaborators will be acknowledged as co-authors and will be expected to provide input to the draft report before its submission to ERG. Potential for publication in peered-review journal will be discussed collectively by the group. The data will only be used for the purpose detailed here and in the analytical plan, any other future analyses in which the contributed data might be pooled will need to be formally agreed upon all contributing parties.

### Ethical issues

The IPD meta-analysis is exempt from ethics approval because we will be collecting and synthesizing data from previous studies in which informed consent had already been obtained, and this meta-analysis will be addressing very similar questions to the research question for which the data were collected (and to which patients gave consent). Moreover, we will request investigators only to submit de-identified datasets.

References

1. Dellicour S, Tatem AJ, Guerra CA, Snow RW, ter Kuile FO. Quantifying the number of pregnancies at risk of malaria in 2007: a demographic study. PLoS medicine. 2010;7(1):e1000221. Epub 2010/02/04. doi: 10.1371/journal.pmed.1000221. PubMed PMID: 20126256; PubMed Central PMCID: PMC2811150.

2. Desai M, ter Kuile FO, Nosten F, McGready R, Asamoa K, Brabin B, et al. Epidemiology and burden of malaria in pregnancy. Lancet Infect Dis. 2007;7(2):93-104. Epub 2007/01/26. doi: S1473-3099(07)70021-X [pii].

3. McGready R, Lee SJ, Wiladphaingern J, Ashley EA, Rijken MJ, Boel M, et al. Adverse effects of falciparum and vivax malaria and the safety of antimalarial treatment in early pregnancy: a population-based study. Lancet Infect Dis. 2012;12(5):388-96. Epub 2011/12/16. doi: 10.1016/S1473-3099(11)70339-5.

4. Walker PG, Ter Kuile FO, Garske T, Menendez C, Ghani AC. Estimated risk of placental infection and low birthweight attributable to Plasmodium falciparum malaria in Africa in 2010: a modelling study. Lancet Glob Health. 2014;2(8):e460-e7. Epub 2014/08/12. doi: S2214-109X(14)70256-6 [pii]

5. WHO. Guidelines for the treatment of malaria. Third edition. Geneva, Switzerland: World Health Organization, 2015.

6. Clark RL. Embryotoxicity of the artemisinin antimalarials and potential consequences for use in women in the first trimester. Reprod Toxicol. 2009;28(3):285-96. Epub 2009/05/19. doi: 10.1016/j.reprotox.2009.05.002.

7. WHO, TDR. Assessment of the safety of artemisinin compounds in pregnancy: report of two joint informal consultations convened in 2006. Geneva: WHO, 2006 WHO/GMP/TDR/Artemisinin/07.1.

8. Finaurini S, Ronzoni L, Colancecco A, Cattaneo A, Cappellini MD, Ward SA, et al. Selective toxicity of dihydroartemisinin on human CD34+ erythroid cell differentiation. Toxicology. 2010;276(2):128-34. Epub 2010/08/07. doi: 10.1016/j.tox.2010.07.016.

9. Dellicour S, Hall S, Chandramohan D, Greenwood B. The safety of artemisinins during pregnancy: a pressing question. Malar J. 2007;6:15. Epub 2007/02/16. doi: 1475-2875-6-15 [pii].

10. Nosten F, McGready R, Alessandro U, Bonell A, Verhoeff F, Menendez C, et al. Antimalarial Drugs in Pregnancy: A Review. Current Drug Safety 2006;1:1-15.

11. Ward SA, Sevene EJ, Hastings IM, Nosten F, McGready R. Antimalarial drugs and pregnancy: safety, pharmacokinetics, and pharmacovigilance. Lancet Infect Dis. 2007;7(2):136-44. Epub 2007/01/26. doi: S1473-3099(07)70025-7 [pii].

12. WHO, TDR. Assessment of the safety of artemisinin compounds in pregnancy: report of two joint informal consultations convened in 2006. Geneva: WHO, 2007 WHO/GMP/TDR/Artemisinin/07.1.

13. Scheuerle A, Tilson H. Birth defect classification by organ system: a novel approach to heighten teratogenic signalling in a pregnancy registry. Pharmacoepidemiology and drug safety. 2002;11(6):465-75. doi: 10.1002/pds.726. PubMed PMID: 12426931.

14. Tinto H, Sevene E, Dellicour S, Macete E, d’Alessandro U, ter Kuile FO, et al. Assessment of the Safety of Antimalarial Drug Use during Early Pregnancy: protocol for a multicenter prospective cohort study in Burkina Faso, Kenya and Mozambique. Reproductive Health. 2015;12:112.

15. Wells GA, Shea B, O’Connell D, Peterson J, Welch V, Losos M, et al. The Newcastle-Ottawa Scale (NOS) for assessing the quality if nonrandomized studies in meta-analyses [May 2015]. Available from: <http://www.ohri.ca/programs/clinical_epidemiology/oxford.htm>.

16. Howards PP, Hertz-Picciotto I, Poole C. Conditions for bias from differential left truncation. Am J Epidemiol. 2007;165(4):444-52. Epub 2006/12/08. doi: kwk027 [pii]. PubMed PMID: 17150983.

17. Margulis AV, Mittleman MA, Glynn RJ, Holmes LB, Hernandez-Diaz S. Effects of gestational age at enrollment in pregnancy exposure registries. Pharmacoepidemiology and drug safety. 2015;24(4):343-52. doi: 10.1002/pds.3731. PubMed PMID: 25702683.

| Table A. Data elements for the individual patient data analysis | |
| --- | --- |
| Variable | **Definition/Description** |
| ID | Unique study identifying number for pregnancy |
| Study Site | Study site identification |
| Enrolment Date | Date of enrolment |
| Age | Maternal age at study enrolment (in years) |
| Enrolment Gestation | Gestational age at enrolment (weeks) |
| Education | Number of years of education |
| Marital | Marital status at time of enrolment |
| Occupation | Main source of income/livelihood |
| LMP | First day of last menstrual period (date) |
| Gravidity | Total number of times that a woman has been pregnant (not including current pregnancy) |
| Prev_Stillbirths | Number of previous stillbirths |
| Prev_Miscarriages | Number of previous miscarriages (including spontaneous and induced abortions) |
| IPTp_SP | Any use of IPTp during index pregnancy |
| IPTp_doses | Number of doses of IPTp during index pregnancy |
| HIV | HIV status |
| Concomitant therapies | Other drugs prescribed to participant |
| Epilepsy | Has the participant been diagnosed with epilepsy? |
| Diabetes | Does the participant have diabetes? |
| TB | Does the participant have TB? |
| Syphilis | Has the participant been diagnosed with syphilis? |
| Smoking | Did the participant smoke during the index pregnancy |
| Alcohol | Did the participant drink alcohol during the index pregnancy |
| ACT | Did the patient have ANY ACT use during index pregnancy |
| Antimalarial name | Name of antimalarial for each treatment |
| Antimalarial start date/ gestational age | Start date of ACT use for each treatment |
| Antimalarial route | Route of administration of antimalarial |
| Antimalarial Confirmed | Source of information on antimalarial use/exposure and/or whether it was confirmed by multiple data source |
| Multiple gestation | Number of fetuses pregnancy during pregnancy |
| Loss to follow up | Was the participant loss to follow up before the end of pregnancy |
| Date of last visit | Last study visit date |
| Date of End of pregnancy | Date of delivery or end of pregnancy |
| Pregnancy Outcome | Pregnancy outcome defined as: Livebirth (including Neonatal Deaths); Miscarriage (confirmed pregnancy that fails to progress, resulting in death and expulsion of the embryo or unviable fetus gestational age < 28 weeks); Stillbirth (confirmed pregnancy the results in birth of stillborn infant gestational age ≥ 28 weeks); Induced Abortion |
| Gestation End Pregnancy | Gestational Age at Birth/End of pregnancy in weeks with 2 decimal |
| Newborn exam age | Age of infant (in days) at the time of the newborn exam |
| Newborn Abnormality | Was any congenital malformations detected following examination of baby? |
| Abnormality description | Description of abnormality was detected |
