# Supplementary material for: First-trimester artemisinin derivatives and quinine treatments and the risk of adverse pregnancy outcomes in Africa and Asia: A meta-analysis of observational studies
Source: PLoS Med. 2017 May 2;14(5):e1002290. doi: 10.1371/journal.pmed.1002290 (PMC5412992; doi:10.1371/journal.pmed.1002290)
Supplement: S2 Text — (DOCX) [file pmed.1002290.s013.docx]

| S2 Text | Characteristics of studies in the individual patient data meta-analysis |
| --- | --- |

| **Author/Principal Investigator** | Christine Manyando |
| --- | --- |
| **Country** | Zambia |
| **Study Period** | October 2004 to July 2008 |
| **Study Design** | Multi-centre, prospective cohort study |
| **Participants** | Pregnant women presenting for antenatal care (ANC) at four clinics |
| **Inclusion and Exclusion criteria** | Pregnant women were eligible for inclusion if they had received AL or SP for the treatment of malaria |
| **Exposure ascertainment** | Self-report through enrolment at ANC and exposure were verified by documentation from their outpatient clinic files |
| **Gestational age measurements** | Last menstrual period date and ultrasound for a few cases or Dubowitz assessment if LMP was unavailable |
| **Follow up visits** | - Women visited the antenatal clinic for assessment of safety parameters at baseline/enrolment, four weeks post-enrolment, four weeks pre-delivery, at delivery, and at six weeks post-delivery. - Infants were followed up at six weeks, 14 weeks, and at 12 months after birth. |
| **Outcome** | - The primary endpoint was the incidence of perinatal mortality (stillbirth or neonatal death within 7 days of birth). - Secondary outcome measures were gestational age at delivery and birth weight adjusted for gestational age. - Exploratory endpoints were assessed: frequency of spontaneous abortion, preterm delivery, neonatal mortality, maternal mortality, major and minor birth defects, and infant development |
| **Statistical Analysis** | - Descriptive data only |
| **Ethical review** | The study protocol was approved by the local Ethics Review Committee of the Tropical Diseases Research Centre, Zambia, and WHO Ethics Review Committee, Geneva. All participants, or their parent/guardian (if the subject was a minor), gave written or finger-marked informed consent before study entry. |
| **Notes** | - Malaria diagnosis was clinically or parasitologically confirmed: malaria was unconfirmed in 82.0% of the AL and 87.2% of the SP exposure groups - First trimester in the paper was defined as 2-12weeks post LMP (exclusive). - SP was the standard anti-malarial treatment during pregnancy at time of study. - Mean gestational age at enrolment was 24.6 weeks (SD 8.01) which is reflected by the low number of miscarriages detected (1% of pregnancies) - 15 miscarriages reported in the manuscript represent 12 pregnancies (1 triplet and 1 twin pregnancy) - Approximately 30% of women in the AL group and 38% in the SP exposure group were tested for HIV - No information on marital status |
| **Bias assessment** | Overall low risk of bias (6/9 stars)   - Selection: 3 /4 stars - enrolled from 4 ANC clinics - Comparability: 1 /2 stars - confounding by indication possible as not randomized/blinded allocation of treatment. SP comparator but not recommended in first trimester and declining efficacy - Outcome assessment: 3 /4 stars - outcome was ascertain prospectively. Attrition was low: 4% (22/495) discontinued in AL exposure group and 6% (28/506) in SP exposure group before the end of pregnancy (14% and 18% by end of study respectively). Relatively late recruitment at ANC means only late miscarriage were captured. |
| **Citation** | Manyando C, Mkandawire R, Puma L, et al. Safety of artemether-lumefantrine in pregnant women with malaria: results of a prospective cohort study in Zambia. Malar J 2010; 9: 249 |

| **Author/Principal Investigator** | Stephen Rulisa |
| --- | --- |
| **Country** | Rwanda |
| **Study Period** | June 2007–July 2009 |
| **Study Design** | Multi-centre (10 health facilities), prospective cohort study |
| **Participants** | Pregnant women presenting for treatment at selected health facilities |
| **Inclusion and Exclusion criteria** | Pregnant women above the age of 18 years were included in the study if the woman was to be treated with AL after diagnosis of uncomplicated *P. falciparum* malaria. A woman with a similar stage of pregnancy and without history of previous or current treatment with AL in the existing pregnancy was selected at the same health centre during routine attendance at the antenatal clinic and invited to participate in the study as part of the control group. |
| **Exposure ascertainment** | - “prospective” ascertainment: women enrolled immediately after prescription of AL for malaria - “Retrospective” ascertainment: women who, during antenatal clinic attendance, were found to have been treated with AL during that pregnancy if treatment could be verified from the patient prescription and treatment register at the health centre. - unexposed group consisted of pregnant women with no history of previous or current treatment with AL in the existing pregnancy and without any signs or symptoms of malaria |
| **Gestational age measurements** | Last menstruation, fundal height and date of quickening were recorded and correlation of at least two of the three factors was used for the gestational age determination. Gestational age was verified by ultrasound in a subset of subjects. |
| **Follow up visits** | Monthly antenatal clinic visits and upon any other visits to the health centre for health concerns, until delivery |
| **Outcome** | - adverse obstetric outcomes: abortion, peri-natal mortality, stillbirth, pre-term delivery, and unexplained neonatal death ≤7 days after birth - adverse infant outcomes: congenital malformations regardless of the pregnancy outcome, and neurological problems |
| **Statistical Analysis** | - Descriptive data only for 1^st^ trimester treatments |
| **Ethical review** | The study was approved by the Rwandan National Ethics Committee prior to commencement. Each patient gave written informed consent before entry into the study. |
| **Notes** | - Some women were enrolled after delivery and were excluded from the IPD analysis (n=288+7) |
| **Bias assessment** | Overall low risk of bias (6/9 stars)   - Selection: 3 /4 stars – enrolled from 10 health facilities - Comparability: 1 /2 stars – the distinction between the effects of malaria and AL exposure could not be made in this study (confounding by indication possible as unexposed not treated for malaria). Unexposed recruited from same population contemporaneously. - Outcome assessment: 3 /4 stars – outcome was ascertain prospectively and retrospectively. Attrition was low: 20 women out of 2070 without complete data. Relatively late recruitment at ANC means only late miscarriage were captured. |
| **Citation** | Rulisa S, Kaligirwa N, Agaba S, Karema C, Mens PF, de Vries PJ. Pharmacovigilance of artemether-lumefantrine in pregnant women followed until delivery in Rwanda. Malar J 2012; 11: 225 |

| **Author/Principal Investigator** | Dominque Mosha |
| --- | --- |
| **Country** | Tanzania |
| **Study Period** | April 2012-March 2013 |
| **Study Design** | Multi-centre, prospective cohort study |
| **Participants** | Pregnant women recruited from 22 Maternal Health clinics or from monthly house visits via demographic surveillance in the two HDSS sites |
| **Inclusion and Exclusion criteria** | Pregnant women with gestational age <20 weeks recruited from Reproductive and Child Health (RCH) clinic during their routine ANC visits and from the community through monthly round-based house visits and through routine HDSS quarterly census. |
| **Exposure ascertainment** | Self-report at ANC and verified by assessing patient’s medical log in the attended health facility, prescription sheet and maternal RCH card. |
| **Gestational age measurements** | LMP or fundal height examination, when the LMP was unknown |
| **Follow up visits** | Women were followed on monthly basis until delivery to monitor pregnancy and birth outcomes. |
| **Outcome** | - Pregnancy Outcomes: maternal mortality, spontaneous abortion (pregnancy lose ≤ 28 weeks of gestation), stillbirth and live birth. - Birth outcome included birth weight, maturity status at birth and presence of congenital anomalies (under the guidance of a specific developed checklist). |
| **Statistical analysis** | Logistic regression model were used to estimate the odds ratio for the association between pregnancy outcomes and first trimester antimalarial exposure. Multivariable model adjusted for age and parity. |
| **Ethical review** | Ethical approval was granted by the Ifakara Health Institute (IHI) ethical review board and the National Institute for Medical Research (NIMR) ethical committee. Written informed consent was obtained from all participants. |
| **Notes** | Issues with recording of LMP- manually derived gestational age at time of exposure but gestational age at time of exit was unreliable |
| **Bias assessment** | Overall low risk of bias (6/9 stars)   - Selection: 3 /4 stars - enrolled from 22 ANC clinics and 20% from community - Comparability: 1 /2 stars - confounding by indication possible as not randomized/blinded allocation of treatment. Unexposed recruited from same population contemporaneously. - Outcome assessment: 3 /4 stars - outcome was ascertain prospectively. Attrition was low: 2167 pregnant women were recruited and 1783 (82.3%) completed the study until delivery. Relatively late recruitment at ANC means only late miscarriage were captured. |
| **Citation** | Mosha D, Mazuguni F, Mrema S, Sevene E, Abdulla S, Genton B. Safety of artemether-lumefantrine exposure in first trimester of pregnancy: an observational cohort. Malar J 2014; 13(1): 197 |

| **Author/Principal Investigator** | Stephanie Dellicour |
| --- | --- |
| **Country** | Kenya |
| **Study Period** | February 2011- December 2013 |
| **Study Design** | Prospective cohort study, part of multi-country ASAP protocol |
| **Participants** | Women of childbearing age (15-49 years) under enhanced morbidity surveillance |
| **Inclusion and Exclusion criteria** | Women between 15 and 49 years of age and active participants of an ongoing morbidity surveillance program under HDSS. Exclusion criteria included: inability to give written informed consent or provide an accurate medical history. |
| **Exposure ascertainment** | Drug exposure data were captured using three approaches:   - interviews with pregnant women visiting the antenatal clinic in referral health facility and at the time of pregnancy outcome follow-up; - record linkage to data on drugs prescribed to WOCBA at the outpatient department in Lwak Hospital - weekly to twice monthly home visits by fieldworkers as part ongoing morbidity surveillance program |
| **Gestational age measurements** | LMP; ultrasound; fundal height and Ballard Score- assessment based on most accurate measure available |
| **Follow up visits** | Through the recommended ANC visit schedule and after pregnancy outcome |
| **Outcome** | Pregnancy outcomes captured at health facility or at home included: miscarriages, stillbirths, live-births, and major congenital malformations detectable at birth by surface examination. |
| **Statistical analysis** | Cox proportional hazard model was used to estimate the hazard ratio for the association between miscarriage and first trimester antimalarial exposure. Multivariable model adjusted for age, occupation and HIV. |
| **Ethical review** | The EMEP study was approved by the ethics committees and institutional review boards of CDC, KEMRI the Liverpool School of Tropical Medicine and the Institutional Review Board of the University of Washington. Written informed consent or assent was obtained from each participant. |
| **Notes** | Low agreement between data sources through record linkage, a high number of unconfirmed first trimester exposures and inability to assess the effect of the number of exposures on outcome |
| **Bias assessment** | Overall low risk of bias (7/9 stars)   - Selection: 4 /4 stars - enrolled women from the community (low refusal rates) - Comparability: 1 /2 stars - confounding by indication possible as not randomized/blinded allocation of treatment. Unexposed recruited from same population contemporaneously. - Outcome assessment: 3 /4 stars - outcome was ascertain prospectively. Attrition was low: 3% (8/299) discontinued in ACT exposure group and 4% (31/835) in unexposed group before the end of pregnancy (3.4% overall). |
| **Citation** | 1. Dellicour S, Desai M, Aol G, Oneko M, Ouma P, et al. (2015) Risks of miscarriage and inadvertent exposure to artemisinin derivatives in the first trimester of pregnancy: a prospective study in western Kenya. Malaria Journal 14: 461. 2. Tinto H, Sevene E, Dellicour S, Macete E, d’Alessandro U, et al. (2015) Assessment of the Safety of Antimalarial Drug Use during Early Pregnancy: protocol for a multicenter prospective cohort study in Burkina Faso, Kenya and Mozambique. Reproductive Health 12: 112. |

| **Author/Principal Investigator** | Esperanca Sevene |
| --- | --- |
| **Country** | Mozambique |
| **Study Period** | September 2011- June 2013 |
| **Study Design** | Prospective cohort study, part of multi-country ASAP protocol |
| **Participants** | Pregnant women identified within the health demographic surveillance system (HDSS) or presenting at ANC (1 health facility) |
| **Inclusion and Exclusion criteria** | - Eligible participants consisted of pregnant women residing in the defined catchment areas, who planned to remain in the study area through delivery and who were willing and able to provide written informed consent. - Exclusion criteria a were refusal to participate or be followed up at the end of pregnancy and any condition that would interfere with the ability to provide informed consent or provide an accurate medical history. |
| **Exposure ascertainment** | The ascertainment of drug exposure was multi-modal and included self-report (prospective and retrospective) and linkage to treatment records at local health facilities, drug prescribing and dispensing clinics. |
| **Gestational age measurements** | LMP; ultrasound; and Ballard Score- assessment based on most accurate measure available |
| **Follow up visits** | Through ANC visits and at delivery |
| **Outcome** | Pregnancy outcomes captured included: late miscarriages, stillbirths, live-births, and major congenital malformations detectable at birth by surface examination. |
| **Statisticial analysis** | Not applicable as no site specific publication |
| **Ethical review** | The protocol was reviewed and approved by the National Bioethics Committee in Mozambique and the Institutional Review Board of the University of Washington. Written informed consent or assent was obtained from each participant. |
| **Notes** |  |
| **Bias assessment** | Overall low risk of bias (6/9 stars)   - Selection: 3 /4 stars - enrolled from 4 ANC clinics - Comparability: 1 /2 stars - confounding by indication possible as not randomized/blinded allocation of treatment. Unexposed recruited from same population contemporaneously. - Outcome assessment: 3 /4 stars - outcome was ascertain prospectively. Attrition was low: 8.0% (2/25) discontinued in ACT exposure group and 4.7% (35/738) in unexposed group before the end of pregnancy. Relatively late recruitment at ANC means only late miscarriage were captured. |
| **Citation** | Tinto H, Sevene E, Dellicour S, Macete E, d’Alessandro U, et al. (2015) Assessment of the Safety of Antimalarial Drug Use during Early Pregnancy: protocol for a multicenter prospective cohort study in Burkina Faso, Kenya and Mozambique. Reproductive Health 12: 112. |

| **Author/Principal Investigator** | Halidou Tinto |
| --- | --- |
| **Country** | Burkina Faso |
| **Study Period** | April 2011- December 2012 |
| **Study Design** | Prospective cohort study, part of multi-country ASAP protocol |
| **Participants** | Pregnant women identified within the health and demographic surveillance system (HDSS) catchment area or presenting at ANC (1 health facility) |
| **Inclusion and Exclusion criteria** | - Eligible participants consisted of pregnant women residing in the defined catchment areas, who planned to remain in the study area through delivery and who were willing and able to provide written informed consent. - Exclusion criteria were refusal to participate or be followed up at the end of pregnancy and any condition that would interfere with the ability to provide informed consent or provide an accurate medical history. |
| **Exposure ascertainment** | The ascertainment of drug exposure was multi-modal and included self-report (prospective and retrospective) and linkage to treatment records at local health facilities, drug prescribing and dispensing clinics. |
| **Gestational age measurements** | LMP; ultrasound; and Ballard Score- assessment based on most accurate measure available |
| **Follow up visits** | Through ANC visits and at delivery |
| **Outcome** | Pregnancy outcomes captured included: late miscarriages, stillbirths, live-births, and major congenital malformations detectable at birth by surface examination. |
| **Statisticial analysis** | Not applicable as no site specific publication |
| **Ethical review** | The protocol was reviewed and approved by the Ethical Review Boards of Centre Muraz Institutional Ethics committee and National Ethics committee in Burkina Faso, and the Institutional Review Board of the University of Washington. Written informed consent or assent was obtained from each participant. |
| **Notes** |  |
| **Bias assessment** | Overall low risk of bias (6/9 stars)   - Selection: 3 /4 stars - enrolled from 4 ANC clinics - Comparability: 1 /2 stars - confounding by indication possible as not randomized/blinded allocation of treatment in first trimester. Unexposed recruited from participant of a randomized controlled trial. - Outcome assessment: 3 /4 stars - outcome was ascertain prospectively. Attrition was low: 0/ 42 discontinued in ACT exposure group and 2.4% (11/672) in unexposed group before the end of pregnancy. Relatively late recruitment at ANC means only late miscarriage were captured. |
| **Citation** | Tinto H, Sevene E, Dellicour S, Macete E, d’Alessandro U, et al. (2015) Assessment of the Safety of Antimalarial Drug Use during Early Pregnancy: protocol for a multicenter prospective cohort study in Burkina Faso, Kenya and Mozambique. Reproductive Health 12: 112. |
