# Supplementary material for: First-trimester artemisinin derivatives and quinine treatments and the risk of adverse pregnancy outcomes in Africa and Asia: A meta-analysis of observational studies
Source: PLoS Med. 2017 May 2;14(5):e1002290. doi: 10.1371/journal.pmed.1002290 (PMC5412992; doi:10.1371/journal.pmed.1002290)
Supplement: S3 Text — (DOCX) [file pmed.1002290.s014.docx]

| S3 Text | Definitions and Exclusion Criteria from SMRU and African sites IPD |
| --- | --- |

#### Definitions

##### Exposures

- Antimalarial first trimester exposure: evidence of an antimalarial exposure between gestational weeks 2-14 exclusive from the date of the last menstrual period. Exposures are considered confirmed if information could be verified across at least two data sources (for example self-reported by the woman and confirmed from the clinic registers).
- Antimalarial exposure in the artemisinin-embryo sensitive period: evidence of an antimalarial exposure between gestational weeks 6-12 inclusive from the date of the last menstrual period.

| *Artemisinins included* | Artemether-lumefantrine, artesunate-amodiaquine (Burkina-Faso only) |
| --- | --- |
| SMRU | - In the SMRU analysis, all women were screened frequently for malaria at SMRU antenatal clinics, all malaria cases were microscopically confirmed, and treatment information was obtained from SMRU antenatal records. - The group with no malaria consisted of women without microscopically confirmed malaria and without antimalarial treatment in the first trimester. - 8/842 (1.0%) quinine treated cases had severe/hyperparasitaemic malaria compared to 37/189 (19.6%) for artemisinins. |
| *Artemisinins included* | Mefloquine-artesunate and Other: artemether-lumefantrine [Coartem], artesunate plus clindamycin, artesunate monotherapy, and dihydroartemisinin-piperaquine. |

##### Outcomes

- Miscarriage was defined as a confirmed pregnancy (identified by pregnancy test or ultrasound and/or examination at antenatal care visit) which ended on or before 28 weeks gestation
- Stillbirth was defined as a confirmed pregnancy lasting until 28 weeks of pregnancy, that resulted in the birth of a stillborn infant
- Major congenital malformation defined as any structural abnormality with surgical, medical or cosmetic importance that is present at birth detected by surface examination.

#### Exclusion criteria for miscarriage analysis

| Africa IPD | - Pregnancies with unknown gestational age; - Pregnancies enrolled at or after 28 weeks gestation or those enrolled at the time or after pregnancy outcome (i.e. only prospective follow up considered); - Pregnancies treated with antimalarials in the first trimester through intravenous or intramuscular route of administration as recommended for severe malaria were not included (proxy for severe malaria); - Pregnancies exposed to either 1) ACT and quinine or 2) who had more than one antimalarial treatment course in the first trimester; - Pregnancies exposed to any antimalarials between 14-18 weeks gestation were excluded from the unexposed group.   *Note: there was no systematic assessment of viability prior to treatment therefore this is unlikely to have affected treatment received in African sites. Data on confirmed malaria infections is not available from African sites, therefore only antimalarial treatment is considered.* |
| --- | --- |
| SMRU | - Women with unknown gestational age; - Women that did not start ANC in first trimester; - Women with only *P.vivax* infection in the first trimester; - Women who had signs of pregnancy loss (clinical or from ultrasound in later years) before antimalarial administration - Quinine exposure group excludes women that received first line quinine followed by an artemisinin due to quinine failure. |

#### Exclusion criteria for stillbirth analysis

| Africa IPD | - Pregnancies with unknown gestational age; - Pregnancies enrolled at time or after pregnancy outcome (i.e. only prospective follow up considered); - Pregnancies treated with antimalarials in the first trimester through intravenous or intramuscular route of administration as recommended for severe malaria were not included (proxy for severe malaria); - Pregnancies exposed to either 1) ACT and quinine or 2) who had more than one antimalarial treatment course in the first trimester; - Pregnancies exposed to any antimalarials between 14-18 weeks gestation were excluded from the unexposed group.   *Note: Pregnancies lost to follow up, ending in miscarriage or induced abortion were included and censored at the time of event or last visit date. Twin pregnancies were included from the African sites.* |
| --- | --- |
| SMRU | - Women with unknown pregnancy outcome; - Women with unknown gestational age; - Women that did not start ANC in first trimester; - Women with only *P.vivax* infection in the first trimester; - Pregnancies enrolled before the introduction of artemisinins in 1994; |

#### Exclusion criteria for major congenital malformations analysis

| Africa IPD | - Pregnancies enrolled at the time or after pregnancy outcome (i.e. only prospective follow up considered); - Pregnancies treated with antimalarials in the first trimester through intravenous or intramuscular route of administration as recommended for severe malaria were not included (proxy for severe malaria); - Pregnancies exposed to either 1) ACT and quinine or 2) who had more than one antimalarial treatment course in the first trimester; - Pregnancies exposed to any antimalarials between 14-18 weeks gestation were excluded from the unexposed group - Pregnancies ending in miscarriage or loss to follow up (unknown pregnancy outcome).   *Note: Twin pregnancies and stillbirths were included from the African sites.* |
| --- | --- |
| SMRU | - Women with unknown pregnancy outcome; - Women with unknown gestational age; - Women that did not start ANC in first trimester; - Women with only *P.vivax* infection in the first trimester; - Women who had a hyperparasitaemic or severe *P. falciparum* infection; - Pregnancies ending in miscarriage ; - Pregnancies enrolled before the introduction of artemisinins in 1994; - Twins |
