# Supplementary material for: First-trimester artemisinin derivatives and quinine treatments and the risk of adverse pregnancy outcomes in Africa and Asia: A meta-analysis of observational studies
Source: PLoS Med. 2017 May 2;14(5):e1002290. doi: 10.1371/journal.pmed.1002290 (PMC5412992; doi:10.1371/journal.pmed.1002290)
Supplement: S4 Text — (DOCX) [file pmed.1002290.s015.docx]

| S4 Text | Statistical analysis methods extended |
| --- | --- |

1. Accounting for left truncation and semi-competing risks:

The investigators arrived at consensus on how to handle and account for left truncation due to different enrolment times of individual women into the cohort study (i.e., contribution of person-time into the study beginning at different gestational weeks). A separate issue, but also in support of our approach was the nature of semi-competing risks. This situation arises in our study where a particular type of event (e.g., miscarriage) can preclude other types of events (e.g., stillbirth, congenital anomaly) from being observed either due to outcome definition (i.e., pregnancy loss post-28 weeks gestation) or possible non-identification/screening of an outcome (i.e., clinical assessment for congenital anomaly at live birth delivery). To address both of these analytic and study design issues, we decided to examine these outcomes separately and not as a composite of adverse pregnancy outcomes. With the time-dependent nature of both the exposures and the pregnancy outcomes of miscarriage, stillbirth and congenital anomaly, not all women were included in the separate analyses. For example, a woman entering the study at gestational week 30 would not be able to contribute person-time to a Cox proportional hazards model relating antimalarial use to the occurrence of miscarriage. We therefore modelled the cause-specific hazards of each outcome in order to estimate the average relative risk due to exposure in the pooled cohort. Inclusion criteria per outcome were assigned for women that were at risk and enrolled (contributing person-time) to each cause-specific Cox proportional hazards model.

1. Time-varying exposure:

Exposure was treated as time-dependant so that participants were considered exposed only from the time they received artemisinin or quinine treatment rather than from the time of enrolment in the study. The unexposed group consisted of a combination of pregnancies never exposed and of pregnancies that were eventually exposed, but d ‘unexposed pregnancy-weeks’ to the unexposed category until the day of exposure, after which they switched to the exposed category. For example if a participant was enrolled in the study at gestational week 8 but received treatment at gestational week 11, she was considered as exposed from week 11 and contributed person-time for the unexposed between weeks 8-11.

1. Model selection:

The crude effect estimates in the IPD analysis were site-adjusted and multivariate analyses were adjusted for potential confounders identified a priori that were available (non-missing and collected in their respective primary studies) from all sites – gravidity and age. We carefully considered other important known confounders, including HIV status and were explored in sensitivity analyses. Approaches such as multiple imputation for missing data were considered but collectively deemed inappropriate because the primary assumptions of data missing at random were violated. HIV as a known confounder was examined in sensitivity analyses. We performed sensitivity analyses with respect to HIV status where estimates were calculated in strata of HIV-positive status known, HIV-negative status known and HIV status unknown, as well as a multivariate analysis that adjusted for HIV status as a categorical variable (positive, negative, missing).

1. Effect modification:

We assessed potential effect modification by our included IPD covariates age (categorically) and gravidity (categorically) each individually using a Wald test while adjusting for other covariates, but none of the interaction terms was statistically significant at p<0.05 and thus the interaction terms were not included in the final models.

We determined that the greatest potential for correlated covariates and interaction existed at the individual level (i.e., where a woman was observed in two separate pregnancies) and across sites. To account for the correlation of women with more than one pregnancy within a study, a robust variance estimator was used. We evaluated effect modification by site in sensitivity analyses of the meta-analysis where one study/site was removed at a time individually, then determining if our pooled results were robust to effects of any one site.

1. Heterogeneity:

Despite low I^2^ values reported in our results (lack of observed excessive heterogeneity), we incorporated clustering on study site into the IPD analyses through site-adjustment. As such, in this individual-level pooled data analysis, we considered the assumption of similar effects (random-effects analysis) to be reasonable versus assuming that our results were observed from a single underlying effect (fixed-effects analysis), particularly when combining the African studies with those data from Thailand.

1. Statistical programs and syntax:
   1. *SAS procedures for 2-stage IPD analyses for miscarriages and stillbirths:*

Listing (A) displays an example SAS statement for the analysis of the IPD Africa data across multiple study sites. We conducted these 2-stage meta-analytic techniques using SAS 9.3 (SAS Institute, Cary NC) via the PROC MIXED procedure. Under the DATA and INFILE statements, we name the dataset *‘ipdmisc’* which is read in from the text file, ‘*ipdmisc.txt’.* As we see under the INPUT command, this data set consists of two columns, *studysite* and *diff*. the first column is the study identification number (from 1 to 4), and the second column are example observed differences of effect sizes for each study.

1. TITLE ‘IPD Miscarriage Outcome’

DATA ipdmisc;

INFILE ‘ipdmisc.txt’;

INPUT studysite diff;

PROC MIXED DATA=ipdmisc;

CLASS studysite;

MODEL diff = / P SOLUTION;

RANDOM studysite / SOLUTION;

REPEATED / GROUP = studysite;

RUN;

The CLASS statement under PROC MIXED specifies categorical variables (i.e., *studysite*) that does not contain quantitative information. The MODEL, RANDOM and REPEATED statements together specified the statistical model we were fitting to the given dataset of women eligible for the outcome (e.g., miscarriage). The MODEL statement identifies our dependent variable and the fixed-effects. In our model, the effect sizes (*diff*) are modeled by the fixed-effects of the interept, which is implied by default. The RANDOM and REPEATED statements together estimate the random effects (*studysite*) and the between-study variance. We requested SAS to include in the output the estimates, standard errors, t-statistics and *P* values for significance testing for each of the fixed (the average overall exposure effect) and random effects using the SOLUTION options.

To determine the amount of heterogeneity between individual study estimates and inform our 1- and 2-stage IPD analyses, we calculated *Q* and *I^2^* statistics using via the PROC MIXED procedure. Detailed steps of our process for calculating statistics to assess heterogeneity are outlined below.

Use of the PROC MIXED and MODEL statements was required, and the RANDOM statement was specified following the MODEL statement. Individual studies’ effect sizes were calculated for fixed and random effects models. Under assumptions that studies differed by sampling error only (homogeneous), then overall effect size from fixed-effects models were considered. When assuming that there were between-studies variability (heterogeneous), then random-effects models were considered to take into account within- and between-studies variability, and overall effect size was determined for a given IPD interpretation. The weight of the individual study results was calculated as proportional to the square of standard errors (B). The weighted average effect size (Theta) was then determined with upper and lower limits given these standard errors (C).

1. Wi=1/(stderr_effectsize^2);
2. Theta=sum(effectsize_i * Wi)/sum(Wi) ;

Std_Theta=sqrt(1/(sumWi));

Upper_Theta=Theta+1.96*Std_Theta;

Lower_Theta=Theat-1.96*Std_Theta;

where i-is the number of the study.

To assess heterogeneity among the studies’ effects, Cochran’s *Q* statistics were calculated (D). *Q* statistic has chi-square distribution with k–1 degree of freedom (k is the number of effect sizes in the sample). High values of Q (above the critical point for a given significance level α) would enable us to reject the null hypothesis about homogeneity and conclude that there is statistically significant variation between studies. Knowing *Q*, the estimator of between-study variance tau can be calculated using Method of Moments (E).

The extent of heterogeneity was estimated by *I^2^* index by comparing the *Q* value with its expected value assuming homogeneity (F). We characterized the proportion of inconsistency in individual studies, where values close to 100% imply very high degrees of heterogeneity. *I^2^* in ranges of 0-40%, 30-60%, 50-90%, and >75% represented low, moderate, high and considerable heterogeneity accordingly. The tests for homogeneity generated output with individual estimates for *Q* and *I^2^* for the interpretation of our results, as well as *Tau^2^* for LS means differences and Cohen’s effect sizes.

1. Q=sum{Wi*(effectsize_i – Theta)^2};
2. If Q > k-1 then tau^2= (Q-(k-1)) / C;

If Q ≤ k-1 then tau^2= 0 ;

Where C = sum (Wi) – (sum (Wi^2))/ (sum(Wi));

1. If Q > k-1 then I^2=100 * (Q-(k-1)) /Q;

If Q ≤ k-1 then I^2= 0;

*6.2 Stata syntax for aggregated data meta-analyses for miscarriages and stillbirths:*

We used the metan command in Stata (v13.1) to perform the aggregate data meta-analysis combining the African IPD and Thai SMRU effect estimates and derive the summary estimate using random effect models using the method of DerSimonian & Laird, with the estimate of heterogeneity being taken from the from the Mantel-Haenszel model, and produced the forest plots as per (A). We considered the assumption of similar effects (random-effects analysis) to be reasonable versus assuming that our results were observed from a single underlying effect (fixed-effects analysis), particularly when combining the African studies with those data from Thai SMRU study.

1. metan LnHR LnLCI LnUCI, eform random effect(Hazard Ratio)

Where LnHR is the natural logarithm of the hazard ratio; LnLCI and LnUCI are the natural logarithms of the lower and upper confidence interval of the hazard ratios respectively.

1. Multivariate meta-analysis:

To evaluate whether our results from cause-specific hazards models evaluating outcomes of miscarriage and stillbirth separately, we performed a post hoc multivariate meta-analysis that allowed us to combine estimates from the related parameters of pregnancy loss (i.e., miscarriage and stillbirth) from each of the separate contributing studies, each with individual-level data for pooling.

We performed our multivariate meta-analyses following the approach of White [White, I. R. 2009. Multivariate random-effects meta-analysis. Stata Journal 9: 40-56.]. By specifying a multivariate regression dataset using a modified ‘mvmeta_make’ program with point estimates, variances and confidence limits from the individual studies’ data, we were able to specify a model estimating risk of miscarriage and stillbirth while still accounting for issues of left truncation using our time-varying Cox model approach above.

Per recommendations from our expert statistical reviewers, this post hoc analysis allowed us to further confirm the direction and confidence in our findings by (i) borrowing strength from other individual studies contributing data and outcomes; (ii) avoid bias from selective outcome reporting; (iii) produce joint confidence/prediction intervals that would potentially increase precision; and (iv) provide an interpretation of coherence with our longitudinal data across multiple studies and outcomes.

- 1. Stata syntax for performing multivariate meta-analysis

mvmeta b V [xvars] [if] [in] [, vars(varlist) wscorr(expression) bscovariance(covtype [matexp]) equations(yvar1:xvar1[, yvar2:xvar2[, ...]]) noconstant commonparm reml ml mm1 mm2 fixed start(expression) longparm noposdef psdcrit(#) maximize_options keepmat(bname Vname) suppress(fe|uv|mm) nouncertainv eform[(name)] dof(expression) randfix[(varlist)] wt[(suboptions)] pbest(min|max [if] [in], pbest_options) print(string) i2 i2fmt( %fmt) ncchi2 ciscale(sd|logsd|logh) testsigma bubble showstart id(varname) showall]
