# Supplementary material for: First-trimester artemisinin derivatives and quinine treatments and the risk of adverse pregnancy outcomes in Africa and Asia: A meta-analysis of observational studies
Source: PLoS Med. 2017 May 2;14(5):e1002290. doi: 10.1371/journal.pmed.1002290 (PMC5412992; doi:10.1371/journal.pmed.1002290)
Supplement: S5 Text — (DOCX) [file pmed.1002290.s016.docx]

| S5 Text | Deviation from published data |
| --- | --- |

The meta-analysis excluded women with multiple antimalarial treatments in the first trimester and excluded women exposed to an antimalarial between weeks 14-18 post LMP to ensure a “purer” unexposed control group.

Manyando et al^37^

The study by Manyando et al in Zambia used a 12 weeks cut-off to define first trimester exposures. Therefore the number of first trimester exposure for Zambia used in the IPD analyses are slightly higher (n=185) compared to the 158 AL exposures reported in the paper. Furthermore, the number of miscarriage differ: the reported number in the published paper was 15 miscarriages however this included a twin and a triplet pregnancy. The IPD analysis counted miscarriage and stillbirth events per pregnancy rather than infants, the number of miscarriage included in the analysis is thus 12.

Rulisa et al^38^

The study enrolled 381 women after pregnancy outcome (19% of total cohort). These retrospective cases were not included in the IPD analysis, therefore the total number of pregnancies included was 1669 rather than the 2050 reported.

Mosha et al^39^

The study by Mosha et al in Tanzania used a 12 weeks cut-off to define first trimester exposures. Therefore the number of first trimester exposure for Tanzania used in the IPD analyses after applying the exclusion criteria described above are slightly different (n=166) compared to the 172 AL exposures reported in the paper.
